# Blood Pressure Changes After a Health Promotion Program Among Mexican Workers

**DOI:** 10.3389/fpubh.2021.683655

**Published:** 2021-06-23

**Authors:** Isabel J. Garcia-Rojas, Negar Omidakhsh, Onyebuchi A. Arah, Niklas Krause

**Affiliations:** ^1^Fielding School of Public Health, Department of Environmental Health Sciences, University of California, Los Angeles, Los Angeles, CA, United States; ^2^Fielding School of Public Health, Department of Epidemiology, University of California, Los Angeles, Los Angeles, CA, United States

**Keywords:** cardiovascular risk factors, intervention study, workplace, health promotion, blood pressure, diabetes mellitus, Mexico, multilevel analysis

## Abstract

**Background:** Cardiovascular disease is becoming increasingly prevalent in low and middle-income countries (LMIC), and high blood pressure (BP) is one of the main risk factors. The efficacy and sustainability of worksite health promotion (WHP) programs for BP reduction in LMIC have yet to be determined.

**Methods:** This non-randomized company-based trial evaluated 6- and 12-months effects of a WHP intervention on BP among 2,002 participating workers from seven Mexican companies. Intervention and control groups were assigned at the company level. The intervention included nutrition counseling, physical exercise, and stress management components. Mixed models assessed differences in BP change between intervention and control companies in intent-to-treat (ITT), per-protocol (PerP), and as-treated (AsTr) analyses, and also within-group changes stratified by company, intervention component, and baseline cardiovascular risk factor levels. All analyses were adjusted for potential confounders. We accounted for missing data and loss to follow-up using inverse probability of censoring weighting.

**Results:** ITT analyses revealed mean BP change differences of −1.1 mmHg at 12 months (95% CI: −2.9; 0.6) in intervention companies relative to control companies. PerP and AsTr analyses confirmed this finding. Within-group analyses showed consistent BP reductions at both 6 and 12 months. Substantial differences in BP changes ranging from diastolic −6.1 mmHg, (95% CI: −11.2; −1.2) to systolic −13.0 mmHg (95% CI: −16.0; −10.1) were found among individuals with diabetes at baseline in intervention companies relative to control companies.

**Conclusion:** After 1 year, WHP was associated with modest but uncertain BP reductions. Substantial reductions were mainly observed among diabetic workers.

## Introduction

Each year, 2.3 million work-related deaths occur worldwide, of which two million are attributable to occupational diseases. Work-related circulatory diseases, including hypertension, are becoming more prevalent in low and middle-income countries (1) and are responsible for 23% of annual work-related deaths globally ranking second after occupational cancer (2).

In Mexico, the top five causes of death in 2017 were all non-communicable diseases, of which circulatory diseases were the greatest contributor (3). High blood pressure (BP) is one of the main risk factors for circulatory diseases, affecting both men and women and contributing to up to 10% of disability adjusted live years lost (3). The costs and financial consequences attributable to hypertension alone increased up to 32% from 2013 to 2018 in several states of Mexico (4), and a recent study indicated that should the prevalence of hypertension in this country remain the same, the number of adults in need of hypertension care will increase 151% by 2050 (5).

Worksite health promotion (WHP) has been identified as advantageous for the management and prevention of non-communicable diseases because work is the place where people spend most of their waking hours and it offers an ideal infrastructure to reach large and captive audiences while eliminating some of the barriers to engage in health promotion activities, such as insufficient time, lack of social support, and limited financial resources (6, 7). Additionally, many benefits have been identified with workplace interventions both at the organizational (reduced absenteeism and health-care costs, increased productivity) and individual (improved morale, increased job satisfaction and health) level (8, 9).

Unfortunately, very few studies reporting the efficacy of workplace interventions come from low and middle-income countries, including Mexico (10). An online search on PubMed, Elsevier, and SciELO using the terms “worksite,” “workplace,” “wellness,” “health promotion,” “interventions,” AND “Mexico” yielded only four longitudinal studies (10–13). These studies had major limitations, including small sample size of self-selected volunteers, lack of a control group, and failure to account for potential confounders or loss to follow-up, and consequently cannot provide reliable evidence for the efficacy of WHP programs in Mexico.

Methodological deficiencies were also noted for studies from high-income countries. A meta-analysis including reviews from the USA, Australia, Canada, Japan, and Europe described available research as being of “suboptimal quality” and inadequate for evaluating sustainability or long-term efficacy (14). A systematic review of wellness programs and their health-related outcomes similarly reported low quality of publications; i.e., no randomized trials or systematic reviews. Only three of the 20 evaluated studies were peer-reviewed, and only one article disclosed a control group to compare intervention participants at the same company (15).

Mexico seeks to meet workers' health needs through social security institutions. The Mexican Institute of Social Security (*Instituto Mexicano del Seguro Social*, IMSS) is responsible for providing universal health insurance to workers in the private sector and their families and is considered the largest social security institution in the country (16).

Aiming to overcome the limitations in research stated above, we evaluated the effects of an IMSS-sponsored multi-component, six-month intervention program on several cardiovascular disease (CVD) risk factors such as BP, body mass index, blood glucose, cholesterol and triglycerides, among employees of diverse companies affiliated with IMSS in Mexico City. This study only reports the effects of the intervention on BP using a control group, long-term follow-up data, and adequate analytic strategies to minimize the threat of bias due to selection and confounding.

## Materials and Methods

### Study Design and Study Population

This prospective, quasi-experimental study consisted of a six-month company-based WHP intervention trial with a 6- and 12-month follow-up after the start of the intervention. IMSS' researchers promoted participation in this study among affiliated companies located in Mexico City and recruited 2,002 workers from seven different worksites, including a cooking utensils factory, a government public health services department, a metalworking company, a pharmaceutical company, a plastic factory, and a printing company. Companies were selected on the basis of their willingness to engage in the study's activities and consented to be part of either a control (*n* = 991) or an intervention group (*n* = 1,011). Employers were presented with the intervention first and if they did not agree to participate, the option to enter the study as a control company was offered second.

### Recruitment of Workers

IMSS researchers met with the directors of each company to introduce the intervention program and obtain authorization to perform the activities. Nurses and social workers from the research team promoted the intervention throughout the company. As an incentive, they offered workers a complete and confidential physical examination, including blood tests such as glucose, cholesterol, and triglyceride levels for free. They remained in each of the companies for about a week to enroll as many participants as possible but no further efforts were made to reach workers on sick leave or disability. All participants provided written, informed consent before the onset of the study.

### Assessment of Baseline Health Status

Individual CVD risk factors were assessed via questionnaires, physical exams, and serologic tests. IMSS experts including occupational physicians, nurses, psychologists, nutritionists, and sports medicine specialists designed a health risk assessment questionnaire assessing socio-demographic and organizational characteristics, behavioral and biological risk factors for CVD, and personal history of diabetes, hypertension, CVD, and other self-reported doctors' diagnoses. The questionnaire was distributed among participants who completed it at home and submitted it to the research team on the day of their physical evaluation.

The physical examination included anthropometric (height, weight, waist circumference, and skinfold measurements to assess body fat, muscle, and bone mass), physiological measurements (heart rate, BP, maximum oxygen intake), and a finger-stick cholesterol and glucose screening (see Supplementary Appendix 1). Assessors were not blind to intervention allocation. Detailed descriptions of these assessments were published previously (17).

### Intervention Program

The WHP program lasted 6 months and included the following components: nutrition counseling, physical activity, and stress management. After initial screening for CVD risk factors through the health risk assessment survey, workers were invited to attend one or more intervention groups according to their specific individual health needs. Participation was voluntary. Prevention activities were offered both at the group and individual level during paid work hours.

#### Nutrition Component

As a first step, workers were invited to participate in one of several offered 30-min information sessions (for maximal 25 participants each) to discuss the basic food groups. Next, two 30-min meetings were held to teach workers how to record their daily food intake and to develop a diet plan according to the workers' preferences and individual needs identified by a licensed and certified nutritionist (Supplementary Appendix 2).

A number of 30 minute individual follow-up sessions were offered based on the World Health Organization classification for body mass index (18): Weekly for obesity class III (BMI ≥ 40), bi-weekly for obesity classes II (BMI 35.0–39.9) and I (BMI 30.0–34.9), monthly for overweight (BMI 25.0–29.9) and underweight (BMI <18.5), and every 2 months for normal weight (BMI 18.5–24.9).

A nutritional history was documented and each worker's diet plan was discussed. Each worker set personal goals and received recommendations to maintain a healthy and balanced diet based on the official Mexican standard 043 (NOM-043-SSA2-2005) and the Eatwell Guide (*Plato del Bien Comer)* (19).

####  Physical Activity Component

Thirty-minute physical exercise sessions were offered daily during the work shift for 24 weeks and were led by a certified group fitness instructor. Each session included warm-up (5 min), aerobics (20 min), and muscle strengthening and stretching (5 min). Workers' workouts were individualized depending on their current physical activity level (determined through the health risk assessment questionnaire) and cardiorespiratory fitness (determined via step test, see Supplementary Appendix 1). Exercise sessions were geared to achieve conditioning responses and optimal benefit according to guidelines from the American College of Sports Medicine (20). Specifically, exercises for sedentary workers (those who did not exercise regularly both at work and off-work) were designed to reach 60% of their maximum heart rate while exercises for active workers (those engaging in regular exercise at least three times per week) were designed to reach 65% of their maximum heart rate. The intensity of exercise was increased by 5% every 4 weeks until participants reached 80% of their maximum heart rate.

#### Stress Management Intervention Component

Weekly sessions of 30 min each, led by a licensed social worker, were offered for groups of 10 to 15 participants on a first-come, first-served basis. These sessions were designed according to secondary prevention stress management programs aiming at the individual with the goal to reduce the severity of stress symptoms before they lead to serious health problems (21). The stress management intervention comprised three different steps, including stress definition and establishment of personal commitments (step 1); redefinition of stress and teaching of stress management techniques (step 2); and follow-up and discussion on how to apply stress management techniques (step 3).

### Health Outcome: Change in BP

At baseline and 6- and 12-month follow-up examinations, BP was measured manually by two research nurses using a sphygmomanometer and following protocols from the American Heart Association (22). Workers rested for about 5 min before the measurement, which was taken on their left arm while sitting. However, only one reading was taken due to time constraints, instead of the two or more consecutive readings recommended by the American Heart Association. Also, inspection of collected BP data revealed a strong terminal digit preference (rounding off readings to the nearest zero value, i.e., the nearest 10 mmHg unit) (23).

In addition to changes in systolic blood pressure (SBP) and diastolic blood pressure (DBP), we also reported changes in pulse pressure (PP) and mean arterial pressure (MAP). Recent evidence suggested PP as a reliable independent predictor of CVD risk and as an important marker of arteriovascular physiopathologic status (24). MAP is the average arterial pressure throughout one cardiac cycle, and it is also known as the steady component of BP (25). MAP is a better predictor for stroke and cerebrovascular events while PP is the main predictor of cardiac events (26). PP was calculated as the difference between SBP—DBP. MAP was calculated as DBP + 0.412^*^(SBP-DBP) (27). BP change since baseline was calculated separately for 6 and 12 months after baseline.

### Potential Confounding Factors

Potentially confounding factors were selected from known risk factors for BP (28–30). We first created a “kitchen sink” regression model including all selected variables and performed backward selection, following recommendations by Vittinghoff et al. (31). Predictors of primary interest (age, gender, and years of education as a proxy for socioeconomic status) and confounding variables important for face validity (personal history of hypertension) were forced into the model. The remaining variables were evaluated one at a time in the full kitchen sink model and those meeting our criterion for selection (i.e., if removal from the model produced an absolute BP change of at least 0.2 mmHg) were retained. The final model included age (years), gender, years of education, personal history of hypertension, alcohol drinking (never; occasionally: ≥ 3 consecutive drinks two to five times per year; frequently: ≥ 3 consecutive drinks at least once per month), body mass index, resting heart rate, LDL cholesterol, job strain ratio, absenteeism days during the year preceding the baseline evaluation, and work shift (morning; accumulated; evening, night, or mixed). Because some continuous variables lack a meaningful zero point, we centered those continuous predictors around the mean value from the sampled subjects (32, 33). Most covariates were collected by the health risk assessment questionnaire and detailed descriptions of their measurement are provided in Supplementary Appendix 1.

### Statistical Analysis

To evaluate the effect of the intervention on the main outcomes, we performed multilevel (linear mixed) analyses, which consider the correlation of repeated measures and combine both random and fixed effects (34). We explored differences between intervention and control companies using intent-to-treat (ITT), per-protocol (PerP), and as-treated (AsTr) analyses. ITT analysis estimated the intervention effect “*as assigned”* and included outcome data for all participants regardless of their adherence to the assigned intervention or missed assessment encounters. In contrast, PerP and AsTr analyses evaluated the effect of the intervention “*as received*” to account for non-adherence. The difference between these two latter methods was the exclusion of non-adherers under the PerP approach (35). In our study, non-adherence was defined as zero participation in any of the intervention components among workers from intervention companies.

To account for loss to follow-up we used *inverse probability of censoring weighting*. In this method, complete cases are weighted by the inverse of their probability of not being censored or lost to follow-up, modeled as function of demographic and other characteristics preceding the timing of the non-loss-to-follow-up. Further, we used a stabilizing factor to normalize the weight (with effect analytical sample size being the size of the observed sample) and to obtain a narrower range of the weight (36). In our study, inverse probability of censoring weighting proved to be a superior method than multiple imputation because most of our missing data were due to non-participation: participants had complete data; non-participants had none (37). Moreover, this method served to avoid fallacious statistical significance due to an inflated sample size (38).

We also investigated within (pre-post) differences among workers who participated in the intervention, separately for its different components. Moreover, we performed secondary analyses to determine if the intervention reached high-risk worker sub-populations: we stratified our mixed models by different baseline risk factor levels of income, body mass index, blood glucose levels, and BP. These analyses are available online as Supplemental Material.

All effect estimates are reported with 95% confidence intervals. For ITT analyses, confidence intervals were bootstrapped using 1,000 draws (39). Data were analyzed using Stata version 14.0.

### Ethics Review and Approval

The study was reviewed and approved by IMSS Institutional Review Board, which has an approved assurance and registration from the Office for Human Research Protections, US Department of Health and Human Services [Department of Health and Human Services, 2009] (registry number IORG0002957). For our study, we also obtained approval from the University of California, Los Angeles (UCLA) Institutional Review Board (IRB#10–000652-CR-00002). The UCLA Institutional Review Board's Federal- wide Assurance with the Department of Health and Human Services is FWA00004642.

## Results

### Study Population Characteristics

**Figure 1:**
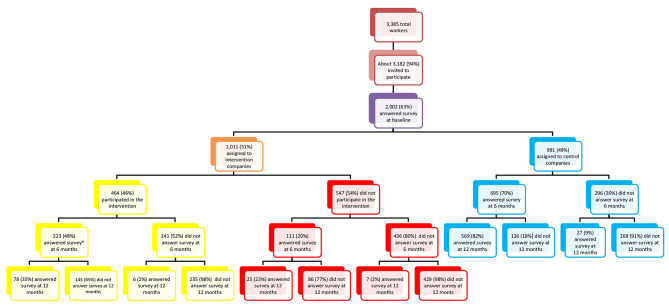
Study population at baseline, 6-month, and 12-month follow-up. Mexican Institute of Social Security study 2009.

Of 3,182 eligible workers in all seven companies who were invited to participate, 2002 (63%) participated in baseline assessments; 51% of participants belonged to intervention companies and 49% to control companies (Figure 1). Companies with the lowest participation rates included the airline company (37.3%) followed by the tire company (54.7%), while the metalworking company and the plastic factory had complete (100%) participation rates. All companies in Mexico are required by law to report an annual medical exam for their workers. The two companies with complete participation rates utilized this study's baseline health risk assessment to comply with such requirement. Worker participation by intervention component is summarized in Supplementary Table A.

Table 1 describes the demographic, biological, behavioral, psychosocial, and work-related characteristics of our sample. Participants were mostly male. The proportion of workers in high-income occupations (defined as above the poverty level for a family of four or > 162,000 Mexican pesos annual income, equivalent to ~8,100 USD) was over 8 times greater in intervention companies and workers in these companies had 4.4 more years of education compared to control companies. Intervention companies were mostly comprised of white-collar employees (professionals, managers, technicians) while control companies had mostly blue-collar (elementary manual labor) workers.

**Table 1:** Characteristics of worker sample in the Mexican Institute of Social Security Study 2009 (*n* = 2,002).

**Variable**	**Intervention companies**		**Control companies**		**Total**	
	***n***	**Frequency (%) or mean (SD)**	***n***	**Frequency (%) or mean (SD)**	***n***	**Frequency (%) or mean (SD)**
**Demographic**						
Gender						
Male	616	60.9	649	65.5	1,265	63.2
Female	395	39.1	342	34.5	737	36.8
Age (years)	1,011	37.5 (10.1)	991	36.1 (11.1)	2,002	36.8 (10.6)
Marital status						
Married	469	46.5	435	43.9	904	45.2
Non-married	540	53.5	555	56.1	1,095	54.8
Education (years)	1,008	14.0 (3.5)	991	9.6 (3.1)	1,999	11.8 (3.9)
Personal annual income (in Mexican pesos)^a^						
Low (<54,000)	175	8.8	658	32.9	833	41.7
Medium (54,001–162,000)	483	24.2	286	14.3	769	38.5
High (>162,000)	352	17.6	45	2.3	397	19.9
**Biological**						
Height (meters)	1,011	1.6 (0.1)	991	1.6 (0.1)	2,002	1.6 (0.1)
Weight (kilograms)	1,011	72.8 (13.8)	991	69.3 (14.1)	2,002	71.1 (14.0)
BMI (kg/m^2^)	1,011	26.9 (4.0)	989	26.7 (4.0)	2,000	26.8 (4.4)
Overweight/obesity^b^						
Yes	677	67.0	605	61.1	1,282	64
No	334	33.0	386	39.0	720	36
Systolic blood pressure (mmHg)	1,011	118.2 (9.9)	991	118.2 (10.7)	2,002	118.2 (10.3)
Diastolic blood pressure (mmHg)	1,011	78.4 (7.1)	991	79.3 (6.6)	2,002	78.8 (6.9)
Hypertension^c^						
Yes	799	79.0	841	84.9	1,640	81.9
No	212	21.0	150	15.1	362	18.1
Resting heart rate (beats/min)	1,001	82.8 (12.2)	988	82.0 (11.2)	1,989	82.4 (11.7)
Blood lipids						
LDL cholesterol (mg/dl)	878	82.7 (29.1)	657	76.3 (30.1)	1,535	79.9 (29.7)
HDL cholesterol (mg/dl)	1,010	39.1 (13.2)	991	34.5 (12.9)	2,001	36.8 (13.3)
Triglycerides (mg/dl)	1,010	139.4 (82.1)	991	142.5 (105.0)	2,001	140.9 (94.1)
Diabetes^d^						
Yes	46	4.6	94	9.5	140	7.0
No	965	95.5	897	90.5	1,862	93.0
**Behavioral**						
Smoking						
Yes	441	43.6	496	50.1	937	46.8
No	570	56.4	495	50.0	1,065	53.2
Leisure-time physical activity (at least 2x/week)						
Yes	269	26.6	222	22.4	491	24.5
No	742	73.4	769	77.6	1,511	75.5
Alcohol drinking (>3 drinks at least 5x/year)						
Yes	837	82.8	724	73.0	1,561	78.0
No	174	17.2	267	27.0	439	22.0
Diet						
Predominantly fruits & vegetables	191	18.9	147	14.8	338	16.9
Predominantly carbohydrates & grains	152	15.0	281	28.4	433	21.6
Predominantly foods of animal origin	668	66.1	563	56.8	1,231	61.5
**Work-related**						
Psychosocial (JCQ score)^e^						
Job strain	1,011	0.8 (0.2)	991	0.9 (0.2)	2,002	0.9 (0.2)
Coworker support	1,011	12.5 (2.0)	991	12.1 (2.2)	2,002	12.3 (2.1)
Supervisor support	1,011	12.4 (2.8)	991	12.5 (2.7)	2,002	12.4 (2.7)
Occupation						
Managers	71	7.0	35	3.5	106	5.3
Professionals	337	33.3	21	2.1	358	17.9
Technicians & associated professionals	186	18.4	55	5.6	241	12.0
Clerical support workers	174	17.2	63	6.4	237	11.8
Service & sales workers	-	-	14	1.4	14	0.7
Craft & related trades workers	56	5.5	82	8.3	138	6.9
Plant & machine operators & assemblers	51	5.0	259	26.1	310	15.5
Elementary occupations (manual labor)	136	13.5	462	46.6	598	29.9
Worksites						
Public Health	123	6.1	-	-	123	6.1
Airline	703	35.1	-	-	703	35.1
Pharmaceutical	185	9.2	-	-	185	9.2
Tools manufacture	-	-	161	8	161	8
Cooking utensils manufacture	-	-	108	5.4	108	5.4
Plastic factory	-	-	95	4.8	95	4.8
Printing company	-	-	627	31.3	627	31.3
Contract type						
Permanent	812	80.6	729	73.6	1,541	77.1
Temporary	196	19.4	261	26.4	457	22.9
Shift						
Morning	771	76.3	441	44.7	1,212	60.7
Evening	26	2.6	21	2.1	47	2.3
Night	13	1.3	5	0.5	18	0.9
Mixed	189	18.7	513	52.0	702	35.2
Double shift	12	1.2	6	0.6	18	0.9
Seniority (years)	1,011	8.6 (9.0)	990	5.2 (6.9)	2,001	6.9 (8.2)
Sick leave (days during year of evaluation)	943	2.6 (11.2)	953	3.2 (13.8)	1,896	2.9 (12.6)
Physical work demands						
Vigorous	85	8.6	261	26.8	346	17.7
Moderate	278	28.2	391	40.1	669	34.1
Light	622	63.2	323	33.1	945	48.2

a $1.00 US dollar ≈ $19.09 MX pesos. As of December 2018, the minimum wage in Mexico was $88.36MX per day ($11.05 per hour) [Banco de Mexico, 2018]. The annual minimum wage is about $22,090.00MX (2,000 working hours/year^*^11.05), which is approximately equivalent to $1,157.15 US dollars.

b *Overweight/obesity determined using the World Health Organization's cutoffs: Body mass index ≥ 25 kg/m^2^.*

c *Determined by self-report and on-site measurement; classified using the American Heart Association (AHA) cutoffs (systolic blood pressure ≥ 130mmHg or diastolic blood pressure ≥ 80 mmHg).*

d *Determined by self-report and on-site measurement; classified using the World Health Organization cutoff ≥ 126 mg/dL.*

e *JCQ, Job Content Questionnaire*.

### Intervention Effects

#### Between-Group Analyses

##### ITT Analyses

Differences in average BP change were small and imprecise at 6 months. At 12 months, differences indicated more substantial BP reductions of around −1 mmHg in intervention companies compared to control companies, after adjusting for potential confounders (Table 2). The maximum differences were observed for SBP (−1.6 mmHg; 95% CI −3.7, 0.6) and MAP (−1.1 mmHg; 95% CI −2.9, 0.6) at 12-month follow-up. Estimates at 12 months were still imprecise, and bootstrapped estimators in general resulted in wider confidence intervals (Supplementary Table B) than confidence intervals derived by robust estimators with inverse probability of censoring weighting.

**Table 2:** Between-group differences in blood pressure change from baseline to 6 and 12 months after intervention.

	**ITT**			
	**Delta^**a**^ crude**	**Delta adjusted^**b**^**	**95% CI**	***p*-value**
Systolic blood pressure (mmHg)				
6 months	0.5	0.2	−1.4, 1.8	0.79
12 months	−1.2	−1.6	−3.7, 0.6	0.16
Diastolic blood pressure (mmHg)				
6 months	0.5	0.0	−1.6, 1.6	0.99
12 months	−0.2	−0.8	−2.3, 0.7	0.31
Pulse pressure (mmHg)				
6 months	0.0	0.2	−0.5, 0.9	0.60
12 months	−1.3	−0.8	−2.0, 0.4	0.18
Mean arterial pressure (mmHg)				
6 months	0.5	0.1	−1.5, 1.7	0.92
12 months	−0.6	−1.1	−2.9, 0.6	0.20

*Intent-to-treat analysis (ITT).*

*Results are based on a mixed-model analysis using inverse probability of censoring weights.*

a *Delta: regression coefficient.*

b *Adjusted for demographic, biological, behavioral, psychosocial, and work-related variables*.

##### PerP and AsTr Analyses

PerP analyses, as seen in Supplementary Table C, comparing workers from intervention companies who participated in any offered intervention sessions with those who did not yielded maximum reductions in SBP and PP (average change of −0.6 mmHg) at 12 months. Effect sizes were up to three-fold smaller compared to ITT analyses. Consistent small BP increases of about +0.2 mmHg were observed in PerP analyses at 6 months. AsTr analyses followed the same pattern but effect sizes were even smaller. Between-group BP changes by specific intervention component are displayed in Supplementary Table D.

#### Within-Group Analyses

Within-group analyses (Supplementary Tables E, F) showed consistent BP reductions, which were more substantial at 12 months (up to−5 mmHg in SBP (95% CI−7.5,−2.6) and−4.8 mmHg in PP (95% CI−8.9,−0.8) for the exercise component).

#### Secondary Analyses Stratified by Selected Baseline CVD Risk Factors

In secondary sub-group analyses by high-risk status (Supplementary Tables G–J), the largest differences in BP reductions were observed for SBP among workers with diabetes at baseline: up to −13.0 mmHg (95% CI −16.0, −10.1) at 12 months follow-up between workers in intervention companies compared to those in control companies (Supplementary Table I). Analyses stratified by hypertension status at baseline showed substantial increases of 5–6 mmHg in MAP in workers with elevated BP at baseline (Supplementary Table J).

## Discussion

### Between-Group Differences in BP Change

ITT analyses revealed only small and imprecise changes post intervention at 6 months but notable differences of about −1 mmHg at 12 months, with a maximum difference of −1.6 mmHg for SBP among employees working in intervention companies compared to those employed in control companies. Similar patterns albeit with smaller effect sizes were observed in PerP and AsTr analyses.

### Are the Observed Modest Intervention Effects Biologically Significant?

Our primary analyses using the recommended ITT approach reveal modest but still substantial BP reductions in intervention companies relative to control companies. We consider a 1-mmHg difference in BP change and actually any difference >0.2 mmHg as substantial for several reasons. First, such changes are comparable to yearly BP changes observed in aging populations. For example, ambulatory SBP among normotensive and treated hypertensive seniors increased 0.4 mmHg per year of age, whereas ambulatory DBP decreased 0.2 mmHg per year of age (40). Moreover, previous epidemiological research has shown that 1 to 2 mmHg reductions in BP at the population level can have a meaningful impact on the incidence of CVD. Specifically, several meta-analyses summarized by Grossman (41) reported that a 1 mmHg SBP reduction decreases the risk of stroke by 5%. Another study, assuming a practical realistic intervention scenario targeted to those with elevated BP, indicated that 1 mmHg reduction was associated with 20.3 and 13.3 fewer heart failure events per 100,000 person-years in African Americans and whites, respectively (42); i.e., a 0.2 mmHg would be associated with 4.1 and 2.7 fewer heart failure events per 100,000 person-years, respectively. Nationwide, this small BP reduction among African Americans and white US populations aged 45 to 64 years would prevent ~1,868 incident heart failure events annually. It is likely that a similar BP reduction would have a greater impact among Hispanic populations as they are generally exposed to a greater number of coronary heart disease risk factors such as lower socioeconomic status, education, and less access to health care (43).

### Comparison With Previous Studies

Our WHP program achieved better results when compared to other studies using an ITT approach. The few recently published, peer-reviewed randomized controlled studies on the effects of multicomponent health promotion programs on BP that used an ITT approach show inconsistent results. A randomized clinical trial of a multiyear, multicomponent workplace wellness program implemented among 32,974 employees at a large US warehouse retail company found that individuals in workplaces where the program was offered reported better health behaviors but neither differences in BP nor other clinical measures of health after 18 months were observed (44). Another large randomized clinical trial among 4,834 university employees found no effects on BP or other clinical health outcomes after a 30-month wellness program (45).

A systematic review of 31 studies between 1980 and 2005 that used an assessment of health risks (including BP) combined with WHP interventions reported a median decrease of −2.6 mmHg SBP and −1.8 mmHg DBP in favor of the intervention using within-group pre-post analyses (46), which is comparable with the lower range of our within-group results. However, our achieved reductions of BP appear smaller compared to clinical interventions among patients in a health-care setting (47).

Since lowering BP is necessary to limit the most serious (including fatal) complications of hypertension, it is important to find alternatives that would reduce the doses of or the need for anti-hypertensive medication. Antihypertensive medication is frequently associated with adverse effects, which may result in non-compliance to treatment and lower quality of life (48, 49). Therefore, primary prevention of modifiable CVD risk factors before manifestation of hypertension or an initial CVD event is preferable to and more effective than cardiac rehabilitation (50), thus making WHP an appealing approach to prevent the onset of morbidities that would require medications.

### Strengths

Strengths of the current study include its large and relatively heterogeneous sample with respect to age, gender, occupation, and industry. The frequency, duration, and content of all components of the intervention have been thoroughly described and this is one of the first intent-to-treat WHP studies in a middle-income country. Also, unlike other multicomponent intervention programs (44), this study was able to explore the separate effects of the different components of this health promotion program. Another strength was the one-year length of follow-up that enabled us to evaluate long-term sustainability of effects.

In addition to SBP and DBP, our study also evaluated PP and MAP. All of our analyses were based considering these four BP components and throughout this paper we mostly reported on SBP and DBP or on consistent overall effects across different BP measures. There were some instances where either SBP or DBP alone would not depict a definite result but when looking at PP and/or MAP a clearer pattern would emerge, particularly in regards to the overall direction of effects (BP reduction or increase).

### Limitations

One important limitation relates to BP measurement. A standardized procedure was not strictly followed as we had only one measurement at a time instead of the two or more consecutive readings recommended by the American Heart Association (22). Additionally, we noted a terminal-digit preference, which may point to insufficient training or supervision of the staff in charge of taking BP measurements, which limited our ability to accurately measure BP changes and most likely led to non-differential misclassification and an underestimation of reported effect sizes.

As with any non-randomized study, non-measured factors could not be controlled and we cannot rule out the possibility that individuals who work in the control companies may be structurally different from those in the intervention companies.

Follow-up data were frequently missing. We addressed this limitation by using a linear mixed model analysis [known for its ability to give unbiased results in the presence of missing data (51)] and applying inverse probability of censoring weighting that accounted for incomplete data (37).

The intervention program might not have been state-of-the-art. Public health knowledge is always evolving and what is considered best practice now may not have been promoted 10 years ago. For example, our nutrition intervention component included dietary recommendations to reduce caloric intake according to gender and general physical activity but did not consider the caloric needs due to occupational physical activity. This is important because even occupations with moderate activity result in a daily energy expenditure of at least 1,680 kcal in an eight-hour shift (52). A better approach could be to change the composition of meals: more protein and less starch and sugar to fulfill workers' caloric needs while improving their CVD risk (53).

Finally, although this population was diverse, results may not generalize to other workplace settings or populations. Participation was voluntary in some companies, which may have introduced selection bias. However, randomization and representativeness in such workplace-based trials can hardly be achieved because it is extremely difficult to randomly recruit workplaces.

## Conclusion

Our primary analyses using the recommended ITT approach revealed differences of about −1 mmHg at 12 months in intervention companies relative to control companies. This finding was consistent with PerP and AsTr analyses. Within-group analyses showed BP reductions at both 6 and 12 months, with effect sizes up to four-fold larger than those found with between-group comparisons. Although individuals with low CVD risk factors at baseline seemed to benefit most from the intervention, people with diabetes who participated in the intervention showed the largest reductions of up to −13.0 mmHg for SBP at 12-months follow-up. However, because BP increases among individuals with Stage II hypertension at baseline were observed, recommendations for this type of intervention need to be made with caution and should take into consideration baseline CVD risk factors. Confirmatory WHP studies targeted to these high-risk populations are warranted.

## Data Availability Statement

The original contributions presented in the study are included in the article/Supplementary Material, further inquiries can be directed to the corresponding author.

## Ethics Statement

The studies involving human participants were reviewed and approved by IMSS Institutional Review Board (IRB), which has an approved assurance and registration from the Office for Human Research Protections, US Department of Health and Human Services [Department of Health and Human Services, 2009] (registry number IORG0002957). For our study, we also obtained approval from the University of California, Los Angeles (UCLA) IRB (IRB#10–000652-CR-00002). The UCLA IRB's Federal- wide Assurance with the Department of Health and Human Services is FWA00004642. Written informed consent to participate in this study was provided by the participants' legal guardian/next of kin.

## Author Contributions

IG-R is the main author and was responsible for the conception, design, data acquisition, analysis, and interpretation for the work. NK and NO along with the main author drafted the work and revised it critically for important intellectual content and participated in the analysis, and interpretation of data. OA provided guidance and valuable insight for the analysis, and interpretation of data for the work. All authors gave the final approval of the version to be published.

## Conflict of Interest

The authors declare that the research was conducted in the absence of any commercial or financial relationships that could be construed as a potential conflict of interest.
