# Supplementary material for: Blood Pressure Changes After a Health Promotion Program Among Mexican Workers
Source: Front Public Health. 2021 Jun 23;9:683655. doi: 10.3389/fpubh.2021.683655 (PMC8261043; doi:10.3389/fpubh.2021.683655)
Supplement: Supplementary file 1 [file Table_1.DOCX]

# Appendix 1. Measurement of anthropometric and physiological indicators

## Measurements

All measurements were performed inside participating companies’ facilities. The following instruments were used: wood bench with two steps, tape measures, chronometer, floor scales with stadiometer, sphygmomanometer, electronic metronome, and computer.

Parameters and measurement procedures are described below:

***Anthropometric Data***

**Weight and height** were measured in kg and cm, respectively, using a floor scale with stadiometer (Salter Brecknell brand). Workers were asked to remove their shoes and wore their usual clothing or uniform. Body mass index was calculated by dividing weight in kilograms by height in meters squared.

**Waist and hip diameter:** A flexible tape measure was used to record abdominal circumference at the navel level or at an intermediate line between the costal border and the iliac crests. Hip circumference was measured at the widest part of the gluteus zone.

***Physiological Data***

**Resting heart rate and blood pressure:** Heart rate was evaluated via auscultation by a research nurse using a 3M™ Littman® Lightweight stethoscope. Blood pressure was measured by two research nurses using a hand-held aneroid sphygmomanometer (DEWIMED® brand) following the auscultatory or Korotkoff method described by the American Heart Association (AHA) [1]. However, only one reading was taken due to time constraints, instead of the two or more consecutive readings recommended by the AHA. Workers rested for about 5 minutes before the measurement, which was taken on their left arm while sitting.

**Submaximum heart rate:** Before determining the submaximum heart rate, auscultation of the heart was performed by an occupational physician using a 3M™ Littman® Lightweight stethoscope to screen for murmurs or arrhythmias indicating a possible heart valve dysfunction or heart disease that would lead to exclusion from the step test.


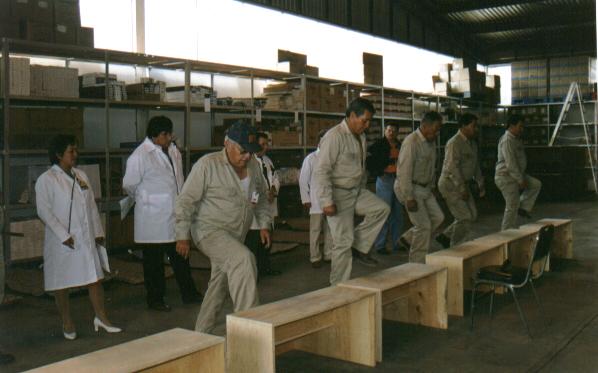
The submaximum heart rate was obtained by applying Manero’s protocol [2], which consisted on stepping up and down an ergometric bench with the following dimensions: 1 meter wide, 50 centimeters high, and 70 centimeters deep. The steps were 25 centimeters high by 35 centimeters wide [3]. The frequency of each ascent was calculated as 90 for active workers and 84 for sedentary workers. One minute training was given to workers before the exercise to explain how to step up the bench (six steps to go up and six to come back down). The test lasted five minutes and was guided by an electronic metronome that marked the frequency of steps. A trained nurse measured each participant’s heart rate by direct auscultation during the first 15 seconds immediately after the five-minute exercise. This value was multiplied by 4 to obtain the sub-maximum heart rate and logged into a software that applied a correction factor by age and made group comparisons in Manero’s nomograph [2], giving automatically as a result the oxygen uptake in liters per minute.

**Screening of glucose, cholesterol, and triglycerides.**

Levels of glucose, cholesterol, and triglycerides were assessed using a CardioChek® Plus analyzer (CCPA, PTS Diagnostics, model 2009). The materials used to take the test were alcohol swabs, sterile lancets, gloves, test strips (depending on the desired test), memochip, and the device CCPA.

The procedure was as follows: patient was asked to rub hands to warm his fingers in order to increase blood flow. Next, the patient was asked to keep his arm down to allow the blood flow to the fingertips. The finger was then cleaned with an alcohol swab and was dried with a gauze pad. After opening the sterile lancet, the patient’s finger was punctured on the side.

To obtain a blood drop for analysis, the patient’s finger was gently pressed, starting at the base and moving towards the tip. The first drop of blood was wiped with sterile gauze and the second drop was used for analysis. The blood was collected with a pipette or capillary that guaranteed that the volume of blood applied to the test strip was adequate. The pipette was completely filled without allowing air bubbles to form. All materials that came into contact with blood were disposed into an infectious biological waste can.

To analyze a blood sample, the memochip corresponding to the batch of strips being used was inserted with the top notch facing up, and the batch number code facing down. After turning on the CCPA device by pressing any button, the test strip was inserted into its corresponding slot. When the display showed “APPLY SAMPLE”, the blood collected in the pipette was applied to the test strip. Results appeared on screen in less than two minutes and were recorded in a registration sheet. The test strip was removed from the analyzer and discarded into an infectious biological waste can.

Performance of the CCPA has been found acceptable when compared to the National Health Laboratory Service methods and conforming to National Cholesterol Education Program guidelines [4, 5]. However, a negative bias and underestimation of risk have also been reported by other researchers evaluating this device [6, 7]

**References**

1. Muntner P, Shimbo D, Carey RM, Charleston JB, Gaillard T, Misra S, et al. Measurement of Blood Pressure in Humans: A Scientific Statement From the American Heart Association. Hypertension. 2019;73(5):e35-e66.

2. Manero R, Armisen A, Manero J. Practical methods to estimate work physical capacity. PAHO Bulletin. 1986;100(2):170-81.

3. Manero R, Manero J. Dos alternativas para el estudio y promoción de la capacidad física de los trabajadores. Mapfre Seguridad. 1991;44:31-7.

4. Panz VR, Raal FJ, Paiker J, Immelman R, Miles H. Performance of the CardioChek PA and Cholestech LDX point-of-care analysers compared to clinical diagnostic laboratory methods for the measurement of lipids. Cardiovascular journal of South Africa : official journal for Southern Africa Cardiac Society [and] South African Society of Cardiac Practitioners. 2005;16(2):112-7.

5. Gao Y, Zhu CG, Wu NQ, Guo YL, Liu G, Dong Q, et al. [Study on the reliability of CardioChek PA for measuring lipid profile]. Beijing da xue xue bao Yi xue ban = Journal of Peking University Health sciences. 2016;48(3):523-8.

6. Park PH, Chege P, Hagedorn IC, Kwena A, Bloomfield GS, Pastakia SD. Assessing the accuracy of a point-of-care analyzer for hyperlipidaemia in western Kenya. Tropical medicine & international health : TM & IH. 2016;21(3):437-44.

7. Whitehead SJ, Ford C, Gama R. The impact of different point-of-care testing lipid analysers on cardiovascular disease risk assessment. Journal of clinical pathology. 2014;67(6):535-9.
