# Supplementary material for: Blood Pressure Changes After a Health Promotion Program Among Mexican Workers
Source: Front Public Health. 2021 Jun 23;9:683655. doi: 10.3389/fpubh.2021.683655 (PMC8261043; doi:10.3389/fpubh.2021.683655)
Supplement: Supplementary file 2 [file Table_2.DOC]

# Appendix 2. Nutrition forms.

**1. Nutrition history**

| Date: /____/_____/____/  Name_________________________________________ ________________________ | Gender: __________  Age: __________ Occupation:_____________________________ | Height:  Current weight:  Expected weight: |  |
| --- | --- | --- | --- |
|  |
|  |
| Recommended diet: _______________________ | Medication(s):__________________________ | People that live with the patient: __________________ | |

Nutrition evaluation

| N^o^ of meals |  |  |  |  |  |
| --- | --- | --- | --- | --- | --- |
| Schedule |  |  |  |  |  |
| Time to eat |  |  |  |  |  |
| Food origin |  |  |  |  |  |
| People that you eat with |  |  |  |  |  |

| Evaluation date |  |  |  |  |  |  |
| --- | --- | --- | --- | --- | --- | --- |
| Weight |  |  |  |  |  |  |
| Skin folds: |  |  |  |  |  |  |
| Biceps |  |  |  |  |  |  |
| Triceps |  |  |  |  |  |  |
| Subscapular |  |  |  |  |  |  |
| Transverse suprailiac |  |  |  |  |  |  |
| Circumferences: |  |  |  |  |  |  |
| Waist |  |  |  |  |  |  |
| Hip |  |  |  |  |  |  |
| Body mass index |  |  |  |  |  |  |
|  |  |  |  |  |  |  |

| Exercise (times/week): | Physical activity: YES ____ NO____  Type: |
| --- | --- |
| Length : / Type : |

If female:

| Nº of pregnancies: | Maximum weight during pregnancy: ____ |
| --- | --- |
| Nº of times patient nursed: |
| Contraception method: | Estrogen therapy: YES ____ NO____  Type: |

Notes: _________________________________________________________________________________________________________________________________________________________________________________________________________________________________

**2. Activity log**

Date: ____/_____/_____/ Patient number: ___/___/___/___/

Name: _______________________________________________________________

Write down below how you distribute your time on a regular day (during the week). Specify the activity, how much time you invest in such activity, and the time of day when you engage in the activity. Include resting time.

Example

| Activity | Length | Time |
| --- | --- | --- |
| Bath and grooming | 45 minutes | 7 - 7:45 a.m. |

| Activity | Length | Time |
| --- | --- | --- |
|  |  |  |
|  |  |  |
|  |  |  |
|  |  |  |
|  |  |  |
|  |  |  |
|  |  |  |
|  |  |  |
|  |  |  |
|  |  |  |
|  |  |  |
|  |  |  |
|  |  |  |
|  |  |  |
|  |  |  |
|  |  |  |
|  |  |  |
|  |  |  |
|  |  |  |
|  |  |  |
|  |  |  |
|  |  |  |
|  |  |  |
|  |  |  |
|  |  |  |
|  |  |  |
|  |  |  |
|  |  |  |
|  |  |  |
|  |  |  |
|  |  |  |
|  |  |  |
|  |  |  |
|  |  |  |

**3. Nutrition log**

Group: /________/ Date:____/_____/_____/ Patient number: ___/___/___/___/

Name:

Monday

| Meal nº | Time | Food (specify the type of food, preparation, and amount eaten) |
| --- | --- | --- |
|  |  |  |
|  |  |  |
|  |  |  |
|  |  |  |
|  |  |  |

Tuesday

| Meal nº | Time | Food (specify the type of food, preparation, and amount eaten) |
| --- | --- | --- |
|  |  |  |
|  |  |  |
|  |  |  |
|  |  |  |
|  |  |  |

Wednesday

| Meal nº | Time | Food (specify the type of food, preparation, and amount eaten) |
| --- | --- | --- |
|  |  |  |
|  |  |  |
|  |  |  |
|  |  |  |
|  |  |  |

Thursday

| Meal nº | Time | Food (specify the type of food, preparation, and amount eaten) |
| --- | --- | --- |
|  |  |  |
|  |  |  |
|  |  |  |
|  |  |  |
|  |  |  |

Friday

| Meal nº | Time | Food (specify the type of food, preparation, and amount eaten) |
| --- | --- | --- |
|  |  |  |
|  |  |  |
|  |  |  |
|  |  |  |
|  |  |  |

Saturday

| Meal nº | Time | Food (specify the type of food, preparation, and amount eaten) |
| --- | --- | --- |
|  |  |  |
|  |  |  |
|  |  |  |
|  |  |  |
|  |  |  |

Sunday

| Meal nº | Time | Food (specify the type of food, preparation, and amount eaten) |
| --- | --- | --- |
|  |  |  |
|  |  |  |
|  |  |  |
|  |  |  |
|  |  |  |

**4. Nutrition guide**
