# Supplementary material for: Blood Pressure Changes After a Health Promotion Program Among Mexican Workers
Source: Front Public Health. 2021 Jun 23;9:683655. doi: 10.3389/fpubh.2021.683655 (PMC8261043; doi:10.3389/fpubh.2021.683655)
Supplement: Supplementary file 3 [file Table_3.DOCX]

# Supplementary material

## Table of contents

1. Participation in Intervention Programs (**Table A**) …………………………………..…... Page 2
2. Between Group Intention-to-treat (ITT) Analyses with Bootstrapped Confidence Interval Estimates (**Table B**) …………………………...………………………………………… Page 3
3. Per-protocol (PerP) and as-treated (AsTr) Analyses (**Tables C** and **D**) ...………………. Page 4
4. Overall Within-Group Analyses and by Intervention Component (**Table E**) ………..…. Page 8
5. Within-group Analyses by Company and Intervention Component (**Table F**) …………. Page 10
6. Between Group and Within-Group Analyses stratified by Levels of Selected Baseline Cardiovascular Disease Risk Factors (**Tables G-J**) ……………………………………... Page 15
   1. Body mass index (**Table G**) ……………………………………………………….... Page 15
   2. Income (**Table H**) ………………………………………………………………….... Page 17
   3. Diabetes (**Table I**) …………………………………………………………………… Page 19
   4. Hypertension (**Table J**) ……………………………………………………………… Page 21
7. Participation Rates by Baseline Levels of Cardiovascular Disease Risk Factors (**Table K**)

…………………………………………………………………………………………….. Page 23

1. Discussion of Supplementary Material …………………………………………………... Page 24
2. References ………………………………………………………………………………... Page 29

**1. Participation in Intervention Programs**

**Table A** summarizes worker participation by intervention component. A third of participants (464) participated in any component of the intervention for an average of 13 sessions (each session was one hour long). Compliance with the different components of the intervention, measured as percentage of offered intervention hours attended, was highest for nutrition (47%), followed by exercise (33%) and stress management (29%).

**Table A.** Worker participation in the offered intervention sessions^a^ by program component, in the Mexican Institute of Social Security Study 2009 (N = 1,011).

|  | **Intervention component** | | | |
| --- | --- | --- | --- | --- |
|  | **Exercise** | **Nutrition** | **Stress management** | **Any component** |
| Number of participants^a^ | 155 | 406 | 201 | 464 |
| Number of one-hour sessions offered^b^ | 55 | 6^c^ | 38 | 99 |
| Minimum number of hours participated | 1 | 1 | 1 | 1 |
| Maximum number of hours participated | 55 | 12 | 38 | 100 |
| Average attendance among intervention program participants (hours) | 18.3 | 2.9 | 10.9 | 13.4 |
| Average attendance among intervention program participants and non-participants (hours) | 2.8 | 1.2 | 2.2 | 6.1 |
| Maximum person-hours if 100% participation | 8,525 | 2,529 | 7,638 | 18,692 |
| Actual person-hours observed | 2,839 | 1,183 | 2,195 | 6,217 |
| Compliance (%) | 33.3 | 46.8 | 28.7 | 33.3 |

^a^There were 111 workers who participated in all three components of the intervention

^b^For nutrition, individual follow-up sessions were planned according to the following groups based on the WHO classification for body mass index:

- Obese class III (BMI ≥ 40): n = 4, # of offered sessions = 24 (weekly)
- Obese class II (BMI 35.0-39.9): n = 11, # of offered sessions = 12 (biweekly)
- Obese class I (BMI 30.0-34.9): n = 62, # of offered sessions = 12 (biweekly)
- Overweight (BMI 25.0-29.9): n = 188, # of offered sessions = 6 (monthly)
- Normal weight (BMI 18.5-24.9): n = 139, # of offered sessions = 3 (bimonthly)
- Underweight (BMI < 18.5): n = 2, # of offered sessions = 6 (monthly)

^c^Weighted average of number of sessions offered = 2529/406 = 6.229

**2. Between Group Intention-to-treat (ITT) Analyses with Bootstrapped Confidence Interval Estimates (Table B)**

**Table B** Between-group differences in blood pressure change from baseline to 6 and 12 months after intervention in the Mexican Institute of Social Security Study (N = 2,002)

|  | **ITT** | | | |
| --- | --- | --- | --- | --- |
|  | **Delta^1^ crude** | **Delta adjusted^2^** | **95% CI^3^** | **p-value^3^** |
| Systolic blood pressure (mmHg) |  |  |  |  |
| 6 months | 0.3 | 0.2 | -1.4, 2.0 | 0.81 |
| 12 months | -1.5 | -1.7 | -4.6, 1.4 | 0.30 |
|  |  |  |  |  |
| Diastolic blood pressure (mmHg) | |  |  |  |
| 6 months | 0.8 | 0.3 | -1.0, 1.3 | 1.00 |
| 12 months | 0.6 | -0.1 | -2.9, 1.3 | 0.46 |
|  |  |  |  |  |
| Pulse pressure (mmHg) |  |  |  |  |
| 6 months | -0.5 | -0.3 | -1.2, 1.6 | 0.79 |
| 12 months | -2.2 | -1.3 | -3.2, 1.8 | 0.52 |
|  |  |  |  |  |
| Mean arterial pressure (mmHg) |  |  |  |  |
| 6 months | 0.7 | 0.3 | -1.0, 1.4 | 0.91 |
| 12 months | -0.2 | -0.9 | -3.3, 1.1 | 0.31 |

Results are based on a mixed-model analysis using (non)censored inverse probability weights.

^1^Delta: regression coefficient

^2^Adjusted for demographic, biological, behavioral, psychosocial, and work-related variables

^3^Bootstrapped bias-corrected 95% confidence intervals and p-values (1000 repetitions)

**3. Per-protocol (PerP) and as-treated (AsTr) Analyses (Supplemental Tables C and D)**

**Table C** displays results comparing workers from intervention companies who participated in any offered intervention sessions with those who did not.

**Table C.** Between-group differences in blood pressure change from baseline to 6 and 12 months after intervention, based on per protocol (PerP) and as-treated (AsTr) analyses in the Mexican Institute of Social Security Study (N = 2,002)

|  | **PerP (n = 1455)** | | | |  | **AsTr (n = 2002)** | | | |
| --- | --- | --- | --- | --- | --- | --- | --- | --- | --- |
|  | **Delta^1^ crude** | **Delta adjusted^2^** | **95% CI^3^** | **p-value** |  | **Delta crude** | **Delta adjusted^2^** | **95% CI^3^** | **p-value** |
| Systolic blood pressure (mmHg) |  |  |  |  |  |  |  |  |  |
| 6 months | -0.4 | 0.2 | -0.6, 1.0 | 0.66 |  | -0.3 | 0.0 | -0.3, 0.3 | 0.98 |
| 12 months | -0.9 | -0.6 | -1.5, 0.2 | 0.15 |  | -0.5 | -0.3 | -0.6, 0.0 | 0.04 |
|  |  |  |  |  |  |  |  |  |  |
| Diastolic blood pressure  (mmHg) | |  |  |  |  |  |  |  |  |
| 6 months | -0.3 | 0.2 | -0.6, 0.9 | 0.67 |  | -0.2 | 0.0 | -0.3, 0.3 | 0.84 |
| 12 months | -0.2 | 0.2 | -0.4, 0.7 | 0.57 |  | -0.1 | 0.0 | -0.3, 0.3 | 0.84 |
|  |  |  |  |  |  |  |  |  |  |
| Pulse pressure  (mmHg) |  |  |  |  |  |  |  |  |  |
| 6 months | -0.1 | 0.2 | 0.0, 0.4 | 0.05 |  | -0.1 | 0.1 | 0.0, 0.1 | 0.00 |
| 12 months | -0.8 | -0.6 | -1.7, 0.4 | 0.25 |  | -0.4 | -0.3 | -0.8, 0.2 | 0.23 |
|  |  |  |  |  |  |  |  |  |  |
| Mean arterial pressure  (mmHg) |  |  |  |  |  |  |  |  |  |
| 6 months | -0.4 | 0.1 | -0.7, 0.9 | 0.80 |  | -0.2 | 0.0 | -0.4, 0.3 | 0.78 |
| 12 months | -0.5 | -0.3 | -0.8, 0.3 | 0.35 |  | -0.2 | -0.2 | -0.4, 0.0 | 0.09 |

Results are based on a mixed-model analysis using inverse probability of (non)censoring weights.

^1^Delta: regression coefficient

^2^Adjusted for demographic, biological, behavioral, psychosocial, and work-related variables.

^3^95% CI = 95% confidence intervals.

Between-group BP changes by specific intervention component based on PerP and AsTr analyses are displayed in **Table D**.

In PerP analyses, adjusted models showed differences of average BP changes of about -1 to -2 mmHg overall. Specifically, for the exercise component, the difference of average BP change ranged from 0 to -2.5 mmHg. Maximum differences were observed for SBP (-2.4 mmHg; 95% CI -4.8, 0.0) and for PP (-2.5 mmHg; 95% CI -4.9, -0.1) at 12 months.

The nutrition component of the intervention was associated with small BP decreases or increases at 6 months. At 12 months, differences in BP ranged from -0.7 to -2.4 mmHg, except for DBP. PP showed the strongest effect (-2.4 mmHg; 95% CI -3.9, -0.8).

The stress component of the intervention consistently reduced BP at both 6 and 12 months. Average differences ranged from -0.4 to -1.9 mmHg and were largest for SBP (-1.5 mmHg; 95% CI -2.3, -0.8 at 6 months and -1.9 mmHg; 95% CI -4.1, 0.3 at 12 months) and MAP (-1.4 mmHg; 95% CI -2.2, -0.5) at 12 months post-intervention. AsTr analyses of individual intervention components resulted in effect sizes up to six-fold smaller than PerP results.

**Table D.** Between-group differences in blood pressure change from baseline to 6 and 12 months after intervention, by intervention component, based on per protocol (PerP) and as-treated (AsTr) analyses in the Mexican Institute of Social Security Study (N = 2,002)

|  | **PerP (n = 1455)** | | |  | **AsTr (n = 2002)** | | |
| --- | --- | --- | --- | --- | --- | --- | --- |
|  | **Delta adjusted^1^** | **95% CI^2^** | **p-value** |  | **Delta adjusted^1^** | **95% CI^2^** | **p-value** |
| **Exercise component (n = 155)** |  |  |  |  |  |  |  |
| Systolic blood pressure (mmHg) |  |  |  |  |  |  |  |
| 6 months | -1.1 | -3.7, 1.5 | 0.42 |  | -0.2 | -0.5, 0.2 | 0.39 |
| 12 months | -2.4 | -4.8, 0.0 | 0.05 |  | -0.3 | -0.6, 0.0 | 0.08 |
|  |  |  |  |  |  |  |  |
| Diastolic blood pressure (mmHg) |  |  |  |  |  |  |  |
| 6 months | -0.3 | -2.2, 1.7 | 0.78 |  | 0.1 | -0.2, 0.3 | 0.67 |
| 12 months | 0.0 | -1.0, 1.1 | 0.94 |  | 0.1 | 0.0, 0.3 | 0.12 |
|  |  |  |  |  |  |  |  |
| Pulse pressure (mmHg) |  |  |  |  |  |  |  |
| 6 months | -0.8 | -2.2, 0.7 | 0.30 |  | -0.2 | -0.4, 0.0 | 0.03 |
| 12 months | -2.5 | -4.9, -0.1 | 0.04 |  | -0.4 | -0.7, 0.0 | 0.03 |
|  |  |  |  |  |  |  |  |
| Mean arterial blood pressure (mmHg) | | | |  |  |  |  |
| 6 months | -0.6 | -2.7, 1.5 | 0.58 |  | 0.0 | -0.3, 0.3 | 0.78 |
| 12 months | -1.0 | -2.2, 0.3 | 0.12 |  | -0.1 | -0.2, 0.1 | 0.53 |
|  |  |  |  |  |  |  |  |
| **Nutrition component (n= 406)** |  |  |  |  |  |  |  |
| Systolic blood pressure (mmHg) |  |  |  |  |  |  |  |
| 6 months | 0.2 | -0.4, 0.9 | 0.52 |  | 0.0 | -0.2, 0.3 | 0.77 |
| 12 months | -2.1 | -3.3, -0.7 | 0.00 |  | -0.8 | -1.3, -0.4 | 0.00 |
|  |  |  |  |  |  |  |  |
| Diastolic blood pressure (mmHg) |  |  |  |  |  |  |  |
| 6 months | -0.2 | -0.8, 0.4 | 0.48 |  | -0.2 | -0.3, 0.0 | 0.00 |
| 12 months | 0.4 | -1.1, 1.9 | 0.59 |  | 0.2 | -0.3, 0.7 | 0.50 |
|  |  |  |  |  |  |  |  |
| Pulse pressure (mmHg) |  |  |  |  |  |  |  |
| 6 months | 0.5 | -0.1, 1.0 | 0.08 |  | 0.2 | 0.0, 0.4 | 0.04 |
| 12 months | -2.4 | -3.9, -0.8 | 0.00 |  | -1.0 | -1.6, -0.3 | 0.00 |

**Table D.** (cont.)

|  | **PerP (n = 1455)** | | | |  | **AsTr (n = 2002)** | | |
| --- | --- | --- | --- | --- | --- | --- | --- | --- |
|  | **Delta adjusted^1^** | **95% CI^2^** | | **p-value** |  | **Delta adjusted^1^** | **95% CI^2^** | **p-value** |
| Mean arterial blood pressure (mmHg) | | | | |  |  |  |  |
| 6 months | -0.1 | -0.7, 0.5 | | 0.79 |  | -0.1 | -0.2, 0.0 | 0.16 |
| 12 months | -0.7 | -1.9, 0.6 | | 0.30 |  | -0.3 | -0.7, 0.2 | 0.22 |
|  |  |  | |  |  |  |  |  |
| **Stress component (N = 201)** |  |  | |  |  |  |  |  |
| Systolic blood pressure (mmHg) |  |  | |  |  |  |  |  |
| 6 months | -1.5 | -2.3, -0.8 | | 0.00 |  | -0.4 | -0.5, -0.2 | 0.00 |
| 12 months | -1.9 | -4.1, 0.3 | | 0.09 |  | -0.4 | -0.8, 0.0 | 0.04 |
| Diastolic blood pressure (mmHg) |  |  | |  |  |  |  |  |
| 6 months | -1.1 | -1.9, -0.2 | | 0.02 |  | -0.2 | -0.3, -0.1 | 0.00 |
| 12 months | -0.9 | -1.9, 0.0 | | 0.06 |  | -0.2 | -0.4, 0.0 | 0.12 |
|  |  |  | |  |  |  |  |  |
| Pulse pressure (mmHg) |  |  | |  |  |  |  |  |
| 6 months | -0.4 | -0.9, 0.2 | | 0.17 |  | 0.1 | -0.2, 0.4 | 0.49 |
| 12 months | -0.9 | -3.8, 1.9 | | 0.53 |  | 0.0 | -0.3, 0.2 | 0.74 |
|  |  |  | |  |  |  |  |  |
| Mean arterial blood pressure (mmHg) | | | | |  |  |  |  |
| 6 months | -1.3 | | -2.0, -0.5 | 0.00 |  | -0.3 | -0.4, -0.1 | 0.00 |
| 12 months | -1.4 | | -2.2, -0.5 | 0.00 |  | -0.3 | -0.4, -0.1 | 0.00 |

Results are based on mixed-model analyses using (non)censored inverse probability weights.

^1^Delta: regression coefficient (adjusted for demographic, biological, behavioral, psychosocial, and work-related variables).

^2^95% CI = 95% confidence intervals.

**4. Overall Within-Group Analyses and by Intervention Component**

Within-group analyses **restricted to intervention companies and stratified by intervention component** (**Table E**) showed BP reductions at 6 months ranging from -0.1 mmHg to -1.4 mmHg, with the exception of DBP and MAP for the stress component, where small increases of +0.3 and +0.1 mmHg, respectively, were observed. At 12 months, substantial reductions were observed of up to -5 mmHg in SBP (95% CI -7.5, -2.6) and -4.8 mmHg in PP (-8.9, -0.8) for the exercise component.

**Table E.** Within-group differences and 95% confidence intervals (CI) for blood pressure change among workers in intervention companies (N = 1011) at 6 and 12 months after the intervention, stratified by intervention components, in the Mexican Institute of Social Security Study (N = 2,002)

|  | **Delta^1^crude** | **Delta adjusted^2^** | **95% CI** | **p-value** |
| --- | --- | --- | --- | --- |
|  |  |  |  |  |
| **Within intervention companies (n = 1011)** |  |  |  |  |
| Systolic blood pressure (mmHg) |  |  |  |  |
| 6 months | -1.3 | -1.4 | -2.4, -0.3 | 0.01 |
| 12 months | -3.2 | -3.3 | -5.4, -1.3 | 0.00 |
| Diastolic blood pressure (mmHg) |  |  |  |  |
| 6 months | -0.4 | -0.2 | -0.9, 0.5 | 0.55 |
| 12 months | -0.7 | -0.7 | -2.0, 0.7 | 0.35 |
| Pulse pressure (mmHg) |  |  |  |  |
| 6 months | -1.0 | -1.2 | -1.5, -0.9 | 0.00 |
| 12 months | -2.7 | -2.7 | -3.4, -2.0 | 0.00 |
| Mean arterial blood pressure (mmHg) |  |  |  |  |
| 6 months | -0.8 | -0.7 | -1.6, 0.2 | 0.11 |
| 12 months | -1.8 | -1.8 | -3.4, -0.1 | 0.04 |
| **Any component (n = 464)** |  |  |  |  |
| Systolic blood pressure (mmHg) |  |  |  |  |
| 6 months | -0.9 | -1.3 | -1.7, -0.8 | 0.00 |
| 12 months | -3.7 | -4.3 | -4.9, -3.6 | 0.00 |
| Diastolic blood pressure (mmHg) |  |  |  |  |
| 6 months | -0.7 | -0.7 | -1.3, -0.1 | 0.03 |
| 12 months | -0.9 | -1.2 | -3.8, 1.5 | 0.39 |
| Pulse pressure (mmHg) |  |  |  |  |
| 6 months | -0.3 | -0.6 | -2.1, 0.9 | 0.42 |
| 12 months | -3.0 | -3.1 | -5.6, -0.6 | 0.02 |
| Mean arterial blood pressure (mmHg) |  |  |  |  |
| 6 months | -0.8 | -0.9 | -1.4, -0.4 | 0.00 |
| 12 months | -2.1 | -2.5 | -4.4, -0.5 | 0.01 |
| **Exercise component (n = 155)** |  |  |  |  |
| Systolic blood pressure (mmHg) |  |  |  |  |
| 6 months | -1.8 | -1.3 | -2.2, -0.3 | 0.01 |
| 12 months | -2.9 | -5.0 | -7.5, -2.6 | 0.00 |
| Diastolic blood pressure (mmHg) |  |  |  |  |
| 6 months | -1.1 | -0.7 | -1.4, 0.0 | 0.05 |
| 12 months | -0.7 | -0.3 | -1.9, 1.3 | 0.72 |
| Pulse pressure (mmHg) |  |  |  |  |
| 6 months | -0.6 | -0.5 | -2.0, 0.9 | 0.46 |
| 12 months | -2.3 | -4.8 | -8.9, -0.8 | 0.02 |
| Mean arterial blood pressure (mmHg) |  |  |  |  |
| 6 months | -1.5 | -1.0 | -1.3, -0.6 | 0.00 |
| 12 months | -1.6 | -2.3 | -2.4, -2.2 | 0.00 |
| **Nutrition component (n= 406)** |  |  |  |  |
| Systolic blood pressure (mmHg) |  |  |  |  |
| 6 months | -0.6 | -0.7 | -1.3, -0.1 | 0.03 |
| 12 months | -3.6 | -4.3 | -6.5, -2.0 | 0.00 |
| Diastolic blood pressure (mmHg) |  |  |  |  |
| 6 months | -0.7 | -0.6 | -1.4, 0.1 | 0.11 |
| 12 months | -0.8 | -1.1 | -4.1, 1.9 | 0.47 |
| Pulse pressure (mmHg) |  |  |  |  |
| 6 months | 0.1 | -0.1 | -0.4, 0.1 | 0.26 |
| 12 months | -3.0 | -3.1 | -3.8, -2.4 | 0.00 |
| Mean arterial blood pressure (mmHg) |  |  |  |  |
| 6 months | -0.7 | -0.7 | -1.4, 0.0 | 0.06 |
| 12 months | -2.0 | -2.4 | -5.3, 0.4 | 0.09 |
| **Stress component (N = 201)** |  |  |  |  |
| Systolic blood pressure (mmHg) |  |  |  |  |
| 6 months | -0.3 | -0.3 | -1.5, 1.0 | 0.68 |
| 12 months | -3.9 | -3.9 | -7.8, 0.0 | 0.05 |
| Diastolic blood pressure (mmHg) |  |  |  |  |
| 6 months | -0.2 | 0.3 | -1.2, 1.8 | 0.66 |
| 12 months | -0.2 | 0.0 | -1.4, 1.4 | 1.00 |
| Pulse pressure (mmHg) |  |  |  |  |
| 6 months | -0.1 | -0.6 | -1.3, 0.2 | 0.14 |
| 12 months | -4.0 | -4.0 | -9.2, 1.2 | 0.14 |
| Mean arterial blood pressure (mmHg) |  |  |  |  |
| 6 months | -0.3 | 0.1 | -1.2, 1.4 | 0.93 |
| 12 months | -1.7 | -1.6 | -2.4, -0.8 | 0.00 |

^1^Delta: regression coefficient

^2^Adjusted for participation in other interventions as well as for demographic, biological, behavioral, psychosocial, and work-related variables.

**5. Within-Group Analyses by Company and Intervention Component (Table F)**

Sensitivity analyses of within-group differences stratified by both company and intervention component (**Table F**) revealed consistent reductions of BP of about -1 mmHg at 6 months and -3 mmHg at 12 months among workers from all companies participating in any component of the intervention. These reductions were highest in the pharmaceutical and public health companies with up to -6.8 mmHg (95% CI -10.9, -2.6) in SBP at 12 months for the stress management component in the public health company.

**Table F.** Within-group differences in blood pressure change among workers in intervention companies (N = 1011) at 6 and12 months after the intervention, by company and intervention component, in the Mexican Institute of Social Security Study (N = 2,002)

|  | **All intervention companies** | | **Airline company^3^** | | **Public Health company** | | **Pharmaceutical company** | |
| --- | --- | --- | --- | --- | --- | --- | --- | --- |
|  | **Delta^1^ crude** | **Delta adjusted^2^** | **Delta^1^ crude** | **Delta adjusted^2^** | **Delta^1^ crude** | **Delta adjusted^2^** | **Delta^1^ crude** | **Delta adjusted^2^** |
|  | **95% CI** | **95% CI** | **95% CI** | **95% CI** | **95% CI** | **95% CI** | **95% CI** | **95% CI** |
| **Any component** |  | **n = 464** |  | **n = 270** |  | **n = 93** |  | **n = 101** |
| Systolic blood pressure (mmHg) |  |  |  |  |  |  |  |  |
| 6 months | -0.9 | -1.3 | -1.1 | -1.4 | -4.4 | -2.5 | 0.7 | -0.6 |
|  | -2.6, 0.7 | -1.7, -0.8 | -3.2, 1.0 | -3.6, 0.8 | -8.1, -0.6 | -6.8, 1.9 | -2.6, 4.0 | -4.0, 2.7 |
| 12 months | -3.7 | -4.3 | NA | NA | -2.2 | -3.9 | -5.6 | -4.8 |
|  | -6.9, -0.5 | -4.9, -3.6 |  |  | -6.1, 1.7 | -9.5, 1.7 | -10.7, -0.5 | -9.5, 0.0 |
|  |  |  |  |  |  |  |  |  |
| Diastolic blood pressure (mmHg) |  |  |  |  |  |  |  |  |
| 6 months | -0.7 | -0.7 | -0.5 | -0.7 | -3.3 | -2.7 | -0.3 | -0.3 |
|  | -1.8, 0.5 | -1.3, -0.1 | -2.0, 1.0 | -2.3, 0.9 | -5.7, -1.0 | -5.8, 0.4 | -2.4, 1.8 | -2.9, 2.2 |
| 12 months | -0.9 | -1.2 | NA | NA | 0.1 | 0.2 | -2.3 | -2.7 |
|  | -3.0, 1.3 | -3.8, 1.5 |  |  | -1.9, 2.0 | -2.4, 2.8 | -5.2, 0.6 | -6.3, 0.9 |
|  |  |  |  |  |  |  |  |  |
| Pulse pressure (mmHg) |  |  |  |  |  |  |  |  |
| 6 months | -0.3 | -0.6 | -0.7 | -0.8 | -1.2 | 0.4 | 0.8 | -0.4 |
|  | -1.0, 0.5 | -2.1, 0.9 | -2.4, 1.1 | -2.7, 1.1 | -5.1, 2.6 | -3.3, 4.0 | -2.3, 3.9 | -3.7, 2.9 |
| 12 months | -3.0 | -3.1 | NA | NA | -2.6 | -4.0 | -3.5 | -1.7 |
|  | -4.2, -1.9 | -5.6, -0.6 |  |  | -6.1, 0.9 | -8.5, 0.4 | -9.0, 1.9 | -4.7, 1.3 |
|  |  |  |  |  |  |  |  |  |
| Mean arterial blood pressure (mmHg) |  |  |  |  |  |  |  |  |
| 6 months | -0.8 | -0.9 | -0.8 | -1.0 | -3.8 | -2.6 | 0.0 | -0.5 |
|  | -2.1, 0.5 | -1.4, -0.4 | -2.3, 0.7 | -2.6, 0.6 | -6.2, -1.4 | -5.8, 0.6 | -2.2, 2.2 | -3.0, 1.9 |
| 12 months | -2.1 | -2.5 | NA | NA | -0.8 | -1.4 | -4.0 | -3.8 |
|  | -4.9, 0.7 | -4.4, -0.5 |  |  | -3.1, 1.5 | -4.9, 2.0 | -7.1, -0.8 | -7.6, 0.1 |

**Table F.** (cont.)

|  | **All intervention companies** | | **Airline company^3^** | | **Public Health company** | | **Pharmaceutical company** | |
| --- | --- | --- | --- | --- | --- | --- | --- | --- |
|  | **Delta^1^ crude** | **Delta adjusted^2^** | **Delta^1^ crude** | **Delta adjusted^2^** | **Delta^1^ crude** | **Delta adjusted^2^** | **Delta^1^ crude** | **Delta adjusted^2^** |
|  | **95% CI** | **95% CI** | **95% CI** | **95% CI** | **95% CI** | **95% CI** | **95% CI** | **95% CI** |
| **Exercise component** |  | **n = 155** |  | **n = 45** |  | **n = 72** |  | **n = 38** |
| Systolic blood pressure (mmHg) |  |  |  |  |  |  |  |  |
| 6 months | -1.8 | -1.3 | -1.3 | -1.4 | -3.9 | -1.8 | 0.8 | 1.2 |
|  | -4.1, 0.6 | -2.2, -0.3 | -5.7, 3.0 | -6.1, 3.2 | -8.2, 0.4 | -7.0, 3.3 | -4.1, 5.7 | -4.2, 6.5 |
| 12 months | -2.9 | -5.0 | NA | NA | -3.0 | -6.1 | -4.0 | -2.8 |
|  | -3.1, -2.6 | -7.5, -2.6 |  |  | -6.8, 0.8 | -11.3, -0.9 | -10.2, 2.3 | -9.5, 3.9 |
|  |  |  |  |  |  |  |  |  |
| Diastolic blood pressure (mmHg) |  |  |  |  |  |  |  |  |
| 6 months | -1.1 | -0.7 | -0.4 | -0.3 | -2.6 | -1.4 | -1.4 | -1.3 |
|  | -2.8, 0.6 | -1.4, 0.0 | -2.8, 2.1 | -2.9, 2.2 | -5.1, -0.2 | -4.9, 2.2 | -5.5, 2.8 | -6.0, 3.3 |
| 12 months | -0.7 | -0.3 | NA | NA | 0.2 | 0.2 | -4.5 | -2.3 |
|  | -3.1, 1.6 | -1.9, 1.3 |  |  | -1.9, 2.2 | -2.6, 2.9 | -11.0, 1.9 | -8.9, 4.3 |
|  |  |  |  |  |  |  |  |  |
| Pulse pressure (mmHg) |  |  |  |  |  |  |  |  |
| 6 months | -0.6 | -0.5 | -1.0 | -1.2 | -1.4 | -0.3 | 3.0 | 2.9 |
|  | -2.3, 1.1 | -2.0, 0.9 | -4.1, 2.1 | -4.5, 2.1 | -5.9, 3.1 | -4.6, 4.0 | -1.1, 7.2 | -1.7, 7.5 |
| 12 months | -2.3 | -4.8 | NA | NA | -3.5 | -6.3 | 1.3 | 0.4 |
|  | -4.5, 0.0 | -8.9, -0.8 |  |  | -7.1, 0.1 | -10.5, -2.1 | -3.2, 5.8 | -4.4, 5.2 |
|  |  |  |  |  |  |  |  |  |
| Mean arterial blood pressure (mmHg) |  |  |  |  |  |  |  |  |
| 6 months | -1.5 | -1.0 | -0.8 | -0.8 | -3.2 | -1.6 | -0.7 | -0.3 |
|  | -3.2, 0.3 | -1.3, -0.6 | -3.8, 2.2 | -4.0, 2.4 | -5.8, -0.6 | -5.3, 2.1 | -4.7, 3.4 | -4.7, 4.2 |
| 12 months | -1.6 | -2.3 | NA | NA | -1.1 | -2.4 | -4.8 | -2.7 |
|  | -3.4, 0.1 | -2.4, -2.2 |  |  | -3.3, 1.2 | -5.7, 1.0 | -10.9, 1.3 | -8.9, 3.5 |

**Table F.** (cont.)

|  | **All intervention companies** | | **Airline company^3^** | | **Public Health company** | | **Pharmaceutical company** | |
| --- | --- | --- | --- | --- | --- | --- | --- | --- |
|  | **Delta^1^ crude** | **Delta adjusted^2^** | **Delta^1^ crude** | **Delta adjusted^2^** | **Delta^1^ crude** | **Delta adjusted^2^** | **Delta^1^ crude** | **Delta adjusted^2^** |
|  | **95% CI** | **95% CI** | **95% CI** | **95% CI** | **95% CI** | **95% CI** | **95% CI** | **95% CI** |
| **Nutrition component** |  | **n = 406** |  | **n = 234** |  | **n = 88** |  | **n = 84** |
| Systolic blood pressure (mmHg) |  |  |  |  |  |  |  |  |
| 6 months | -0.6 | -0.7 | -0.6 | -0.8 | -4.3 | -2.2 | 1.2 | -0.2 |
|  | -2.6, 1.4 | -1.3, -0.1 | -2.9, 1.8 | -3.2, 1.6 | -8.1, -0.6 | -6.6, 2.2 | -2.5, 4.9 | -4.0, 3.6 |
| 12 months | -3.6 | -4.3 | NA | NA | -1.6 | -3.2 | -6.4 | -6.2 |
|  | -7.8, 0.5 | -6.5, -2.0 |  |  | -5.5, 2.2 | -8.8, 2.3 | -11.7, -1.0 | -11.4, -0.9 |
|  |  |  |  |  |  |  |  |  |
| Diastolic blood pressure (mmHg) |  |  |  |  |  |  |  |  |
| 6 months | -0.7 | -0.6 | -0.2 | -0.6 | -3.6 | -2.7 | 0.1 | 0.5 |
|  | -2.2, 0.8 | -1.4, 0.1 | -1.9, 1.5 | -2.4, 1.3 | -6.0, -1.2 | -5.9, 0.5 | -2.3, 2.4 | -2.3, 3.3 |
| 12 months | -0.8 | -1.1 | NA | NA | 0.0 | 0.1 | -2.4 | -2.9 |
|  | -3.1, 1.4 | -4.1, 1.9 |  |  | -2.0, 2.1 | -2.5, 2.7 | -5.6, 0.8 | -6.9, 1.1 |
|  |  |  |  |  |  |  |  |  |
| Pulse pressure (mmHg) |  |  |  |  |  |  |  |  |
| 6 months | 0.1 | -0.1 | -0.3 | -0.3 | -0.9 | 0.6 | 0.9 | -0.6 |
|  | -0.5, 0.8 | -0.4, 0.1 | -2.5, 1.8 | -2.6, 2.0 | -4.8, 3.0 | -3.1, 4.3 | -2.6, 4.4 | -4.3, 3.0 |
| 12 months | -3.0 | -3.1 | NA | NA | -1.9 | -3.3 | -4.2 | -2.9 |
|  | -5.1, -0.9 | -3.8, -2.4 |  |  | -5.4, 1.6 | -7.7, 1.1 | -10.0, 1.6 | -6.2, 0.5 |
|  |  |  |  |  |  |  |  |  |
| Mean arterial blood pressure (mmHg) |  |  |  |  |  |  |  |  |
| 6 months | -0.7 | -0.7 | -0.4 | -0.7 | -4.0 | -2.5 | 0.4 | 0.1 |
|  | -2.4, 1.0 | -1.4, 0.0 | -2.1, 1.3 | -2.5, 1.1 | -6.6, -1.5 | -5.7, 0.7 | -2.1, 2.9 | -2.7, 2.8 |
| 12 months | -2.0 | -2.4 | NA | NA | -0.6 | -1.2 | -4.3 | -4.5 |
|  | -5.3, 1.2 | -5.3, 0.4 |  |  | -2.9, 1.7 | -4.7, 2.3 | -7.7, -0.9 | -8.7, -0.2 |

**Table F.** (cont.)

|  | **All intervention companies** | | **Airline company^3^** | | **Public Health company** | | **Pharmaceutical company** | |
| --- | --- | --- | --- | --- | --- | --- | --- | --- |
|  | **Delta^1^ crude** | **Delta adjusted^2^** | **Delta^1^crude** | **Delta adjusted^2^** | **Delta^1^ crude** | **Delta adjusted^2^** | **Delta^1^crude** | **Delta adjusted^2^** |
|  | **95% CI** | **95% CI** | **95% CI** | **95% CI** | **95% CI** | **95% CI** | **95% CI** | **95% CI** |
| **Stress component** |  | **n = 201** |  | **n = 54** |  | **n = 77** |  | **n = 70** |
| Systolic blood pressure (mmHg) |  |  |  |  |  |  |  |  |
| 6 months | -0.3 | -0.3 | 0.2 | 0.5 | -4.2 | -2.0 | 2.4 | 0.2 |
|  | -3.7, 3.2 | -1.5, 1.0 | -3.5, 4.0 | -3.8, 4.8 | -8.4, -0.1 | -6.9, 3.0 | -1.5, 6.2 | -3.1, 3.6 |
| 12 months | -3.9 | -3.9 | NA | NA | -3.5 | -6.5 | -5.6 | -1.0 |
|  | -5.8, 2.0 | -7.8, 0.0 |  |  | -7.2, 0.3 | -11.6, -1.5 | -12.0, 0.9 | -6.0, 4.0 |
| Diastolic blood pressure (mmHg) |  |  |  |  |  |  |  |  |
| 6 months | -0.2 | 0.3 | 1.1 | 1.6 | -2.6 | -1.3 | 0.0 | 0.0 |
|  | -2.2, 1.9 | -1.2, 1.8 | -1.2, 3.4 | -1.0, 4.1 | -5.0, -0.1 | -4.7, 2.1 | -2.7, 2.7 | -2.9, 2.9 |
| 12 months | -0.2 | 0.0 | NA | NA | 0.2 | 0.2 | -1.5 | -0.8 |
|  | -1.6, 1.2 | -1.4, 1.4 |  |  | -1.8, 2.1 | -2.3, 2.8 | -5.2, 2.3 | -5.3, 3.7 |
| Pulse pressure (mmHg) |  |  |  |  |  |  |  |  |
| 6 months | -0.1 | -0.6 | -0.8 | -1.1 | -1.8 | -0.6 | 2.3 | 0.5 |
|  | -2.2, 2.0 | -1.3, 0.2 | -3.6, 1.9 | -4.1, 2.0 | -6.1, 2.5 | -4.7, 3.6 | -1.0, 5.5 | -2.6, 3.6 |
| 12 months | -4.0 | -4.0 | NA | NA | -4.0 | -6.8 | -4.3 | 0.3 |
|  | -4.6, -3.3 | -9.2, 1.2 |  |  | -7.6, -0.4 | -10.9, -2.6 | -11.8, 3.3 | -2.9, 3.6 |
| Mean arterial blood pressure (mmHg) |  |  |  |  |  |  |  |  |
| 6 months | -0.3 | 0.1 | 0.7 | 1.1 | -3.3 | -1.6 | 0.9 | 0.1 |
|  | -2.8, 2.2 | -1.2, 1.4 | -2.0, 3.4 | -2.0, 4.1 | -5.9, -0.7 | -5.1, 2.0 | -1.9, 3.6 | -2.7, 2.8 |
| 12 months | -1.7 | -1.6 | NA | NA | -1.3 | -2.5 | -3.5 | -1.1 |
|  | -3.5, 0.1 | -2.4, -0.8 |  |  | -3.4, 0.9 | -5.7, 0.7 | -7.2, 0.2 | -5.5, 3.4 |

^1^Delta: regression coefficient

^2^Adjusted for participation in other interventions as well as for demographic, biological, behavioral, psychosocial, and work-related variables.

^3^The airline company went bankrupt and workers were unable to complete the 12-month evaluation, thus values at 12 months are missing

Results are based on a mixed model analysis using (non)censored inverse probability weights

NA = not available

**6. Between Group and Within-Group Fully Adjusted Analyses Stratified by Levels of Selected Baseline Cardiovascular Disease Risk Factors (Tables S7-S10)**

***Body mass index (Table G)***

Between-group ITT analyses

The normal weight group experienced a consistent and sustained difference of mean BP change of about -1.5 mmHg at 6 months and -3 mmHg at 12 months. BP changes among the overweight and obese subgroups were inconsistent, small and imprecise, up to -1.1 mmHg MAP in the overweight group, and up to +1.6 mmHg SBP in the obese group at 6 months.

Within-group analyses

Within-group analyses revealed inconsistent BP changes among workers with normal weight (reductions in SBP and PP and increase or no change in DBP and MAP, respectively). Among overweight workers, consistent reductions in BP were observed of up to -1.4 mmHg (95% CI -1.8, -1.0) and -5.0 mmHg (95% CI -9.2, -0.7) for SBP at 6 and 12 months, respectively, while obese workers experienced a relative BP reduction of -1 mmHg at 6 months and no change at 12 months.

**Table G**. Between and within-group differences in blood pressure from baseline to 6 and 12 months after intervention, stratified by body mass index classification, in the Mexican Institute of Social Security Study (N = 2,002)

|  | **Between-group (ITT)** | | |  | **Within-group (pre-post)** | | |
| --- | --- | --- | --- | --- | --- | --- | --- |
|  | **Delta^1^ adjusted^2^** | | |  | **Delta^1^ adjusted^2^** | | |
|  | **Normal** | **Overweight** | **Obesity** |  | **Normal** | **Overweight** | **Obesity** |
|  | **BMI <25** | **25<BMI<30** | **BMI≥30** |  | **BMI <25** | **25<BMI<30** | **BMI≥30** |
|  | **n = 718** | **n = 885** | **n = 397** |  | **n = 336** | **n = 479** | **n = 196** |
| Systolic blood pressure (mmHg) |  |  |  |  |  |  |  |
| 6 months | -1.5 | -0.1 | 1.6 |  | -1.1 | -1.4 | -1.8 |
|  | -4.3, 1.4 | -2.1, 1.8 | -2.3, 5.5 |  | -3.1, 0.9 | -1.8, -1.0 | -3.0, -0.5 |
| 12 months | -3.6 | -1.1 | 0.9 |  | -1.8 | -5.0 | 0.1 |
|  | -8.4, 1.2 | -5.2, 2.9 | -3.5, 5.4 |  | -5.4, 1.9 | -9.2, -0.7 | -3.8, 4.0 |
|  |  |  |  |  |  |  |  |
| Diastolic blood pressure (mmHg) | |  |  |  |  |  |  |
| 6 months | -1.3 | 0.2 | 0.1 |  | 0.9 | -0.9 | -0.6 |
|  | -4.9, 2.3 | -1.4, 1.7 | -2.7, 2.9 |  | -0.6, 2.4 | -1.4, -0.3 | -0.9, -0.2 |
| 12 months | -1.6 | -1.1 | 0.9 |  | 1.5 | -1.8 | 0.1 |
|  | -4.4, 1.2 | -3.7, 1.5 | -2.0, 3.8 |  | 0.6, 2.4 | -4.3, 0.6 | -0.3, 0.6 |
|  |  |  |  |  |  |  |  |
| Pulse pressure (mmHg) |  |  |  |  |  |  |  |
| 6 months | 0.0 | -0.2 | 1.2 |  | -2.1 | -0.5 | -1.5 |
|  | -1.4, 1.5 | -1.4, 0.9 | -0.7, 3.2 |  | -3.0, -1.1 | -1.0, 0.0 | -2.9, 0.0 |
| 12 months | -1.8 | 0.0 | 0.1 |  | -3.3 | -3.1 | 0.1 |
|  | -5.5, 1.9 | -1.6, 1.5 | -5.3, 5.5 |  | -6.6, 0.0 | -4.9, -1.3 | -3.6, 3.7 |
|  |  |  |  |  |  |  |  |
| Mean arterial pressure (mmHg) |  |  |  |  |  |  |  |
| 6 months | -1.5 | 0.0 | 0.7 |  | 0.0 | -1.1 | -1.1 |
|  | -4.8, 1.8 | -1.6, 1.6 | -2.5, 4.0 |  | -1.7, 1.7 | -1.5, -0.6 | -1.5, -0.6 |
| 12 months | -2.5 | -1.1 | 0.7 |  | 0.2 | -3.1 | 0.0 |
|  | -5.7, 0.8 | -4.3, 2.1 | -1.6, 3.0 |  | -1.4, 1.8 | -6.3, 0.1 | -1.7, 1.7 |

Results are based on a mixed-model analysis using censored inverse probability weights.

^1^Delta: regression coefficient

^2^Adjusted for demographic, biological, behavioral, psychosocial, and work-related variables

***Income (Table H)***

Between-group ITT analyses

Among the low-income group, we observed a -1.5 and -1 mmHg BP difference between workers in intervention companies compared with those in control companies at 6 and 12 months, respectively. In contrast, the medium-income group showed small, positive differences at 6 months followed by negative differences of up to -3.3 mmHg in SBP (95% CI -6.0, -0.7) at 12 months. Lastly, in the high-income group we observed positive differences up to +1.7 mmHg in DBP at 6 months (95% CI -0.8, 4.2).

Within-group analyses

Consistent BP reductions were observed among the three income groups both at 6 and 12 months, with the greatest reductions observed among workers in the mid-income group of up to -3.9 mmHg (95% CI -5.8, -1.9) SBP.

**Table H.** Between and within-group differences in blood pressure change from baseline to 6 and 12 months after intervention, stratified by income level, in the Mexican Institute of Social Security Study (N = 2,002)

|  | **Between-group (ITT)** | | |  | **Within-group (pre-post)** | | |
| --- | --- | --- | --- | --- | --- | --- | --- |
|  | **Delta^1^ adjusted^2^** | | |  | **Delta^1^ adjusted^2^** | | |
|  | **Low**  **(<18K-54K)**  **n = 833** | **Medium (**  **54,001-162K)**  **n = 769** | **High**  **(162,001 - >198K)**  **n = 397** |  | **Low**  **(<18K-54K)**  **n = 175** | **Medium**  **(54,001-162K)**  **n = 483** | **High**  **(162,001 - >198K)**  **n = 352** |
| Systolic blood pressure (mmHg) |  |  |  |  |  |  |  |
| 6 months | -1.5 | 0.5 | 1.0 |  | -3.3 | -1.3 | -0.7 |
|  | -3.9, 0.9 | -2.6, 3.5 | -1.2, 3.2 |  | -6.1, -0.6 | -3.0, 0.5 | -1.0, -0.4 |
| 12 months | -1.2 | -3.3 | 1.2 |  | -3.8 | -3.9 | -2.2 |
|  | -6.5, 4.1 | -6.0, -0.7 | -0.7, 3.2 |  | -8.3, 0.8 | -5.8, -1.9 | -3.0, -1.3 |
|  |  |  |  |  |  |  |  |
| Diastolic blood pressure (mmHg) | |  |  |  |  |  |  |
| 6 months | -1.4 | 0.3 | 1.7 |  | -1.4 | 0.1 | -0.4 |
|  | -3.9, 1.0 | -2.1, 2.7 | -0.8, 4.2 |  | -3.5, 0.8 | -0.5, 0.8 | -1.3, 0.6 |
| 12 months | 0.3 | -2.1 | -0.2 |  | 0.0 | -1.4 | 0.1 |
|  | -2.5, 3.1 | -4.0, -0.1 | -1.3, 0.9 |  | -3.0, 3.0 | -2.6, -0.1 | -0.3, 0.6 |
|  |  |  |  |  |  |  |  |
| Pulse pressure (mmHg) |  |  |  |  |  |  |  |
| 6 months | -0.2 | 0.1 | -0.8 |  | -2.1 | -1.5 | -0.4 |
|  | -1.1, 0.8 | -1.1, 1.3 | -1.4, -0.2 |  | -3.0, -1.2 | -2.6, -0.4 | -1.1, 0.3 |
| 12 months | -1.5 | -1.2 | 1.3 |  | -3.8 | -2.5 | -2.5 |
|  | -4.3, 1.3 | -2.7, 0.2 | -0.2, 2.7 |  | -5.5, -2.0 | -3.3, -1.7 | -3.5, -1.4 |
|  |  |  |  |  |  |  |  |
| Mean arterial pressure (mmHg) |  |  |  |  |  |  |  |
| 6 months | -1.5 | 0.4 | 1.5 |  | -2.2 | -0.4 | -0.5 |
|  | -3.8, 0.9 | -2.2, 3.0 | -0.9, 3.8 |  | -4.5, 0.2 | -1.6, 0.7 | -1.2, 0.2 |
| 12 months | -0.4 | -2.7 | 0.4 |  | -1.6 | -2.5 | -0.8 |
|  | -4.2, 3.5 | -4.9, -0.5 | -0.9, 1.6 |  | -5.2, 2.1 | -4.1, -0.9 | -1.1, -0.5 |

Results are based on a mixed-model analysis using censored inverse probability weights.

^1^Delta: regression coefficient

^2^Adjusted for demographic, biological, behavioral, psychosocial, and work-related variables.

***Diabetes (Table I)***

Between-group ITT analyses

Results differed substantially by diabetes status, especially at 12 months: While the normal glucose group showed consistent BP decreases of up to -2.5 mmHg (95% CI -5.2, 0.2), the pre-diabetes group showed consistent and large BP increases of up to +8.6 mmHg (95% CI 4.3, 12.9), and the diabetes subgroup consistent and large decreases of up to -13.0 mmHg (95% CI -16.0, -10.1) in SBP. These patterns persisted in analyses restricted to the smaller sample of participants who were evaluated at both 6 and 12 months.

Within-group analyses

Workers with normal blood glucose showed consistent BP reductions at both 6 and 12 months of up to -3.5 mmHg in SBP (95% CI -6.0, -1.1) at 12 months. Workers with pre-diabetes showed about -1 mmHg BP reduction from baseline to 6 months followed by BP increases ranging from +1 to +4 mmHg at 12 months. Among the diabetes group, consistent and large BP reductions were observed from baseline to both 6 and 12 months, of up to -14 mmHg in SBP at 12 months (95% CI -16.7, -11.2).

**Table I.** Between and within-group differences in blood pressure change from baseline to 6 and 12 months after intervention, stratified by blood glucose risk levels, in the Mexican Institute of Social Security Study (N = 2,002)

|  | **Between-group (ITT)** | | |  | **Within-group (pre-post)** | | |
| --- | --- | --- | --- | --- | --- | --- | --- |
|  | **Delta^1^ adjusted^2^** | | |  | **Delta^1^ adjusted^2^** | | |
|  | **Normal**  **(<100mg/dl)** | **Prediabetes**  **(100-125mg/dl)** | **Diabetes**  **(>126mg/dl)** |  | **Normal**  **(<100mg/dl)** | **Prediabetes**  **(100-125mg/dl)** | **Diabetes**  **(>126mg/dl)** |
|  | **n = 1402** | **n = 502** | **n = 97** |  | **n = 798** | **n = 182** | **n = 30** |
| Systolic blood pressure  (mmHg) |  |  |  |  |  |  |  |
| 6 months | 0.8 | 0.6 | -1.8 |  | -1.4 | -0.9 | -3.3 |
|  | -1.2, 2.8 | -2.1, 3.2 | -4.4, 0.7 |  | -2.5, -0.3 | -2.5, 0.6 | -6.1, -0.6 |
| 12 months | -2.5 | 8.6 | -13.0 |  | -3.5 | 4.2 | -14.0 |
|  | -5.2, 0.2 | 4.3, 12.9 | -16.0, -10.1 |  | -6.0, -1.1 | 1.1, 7.2 | -16.7, -11.2 |
|  |  |  |  |  |  |  |  |
| Diastolic blood pressure  (mmHg) | |  |  |  |  |  |  |
| 6 months | 0.4 | -1.0 | 1.8 |  | -0.1 | -0.5 | -2.7 |
|  | -1.5, 2.4 | -2.9, 0.9 | -0.7, 4.3 |  | -0.7, 0.6 | -2.0, 1.1 | -4.6, 0.9 |
| 12 months | -1.1 | 1.3 | -6.1 |  | -0.5 | 0.8 | -10.4 |
|  | -3.1, 0.9 | -2.6, 5.2 | -11.0, -1.2 |  | -1.9, 0.8 | -3.1, 4.8 | -16.9, -3.8 |
|  |  |  |  |  |  |  |  |
| Pulse pressure  (mmHg) |  |  |  |  |  |  |  |
| 6 months | 0.4 | 1.4 | -3.3 |  | -1.4 | -0.6 | -0.2 |
|  | -0.5, 1.3 | 0.1, 2.8 | -7.4, 0.8 |  | -1.8, -0.9 | -1.4, 0.3 | -4.7, 4.2 |
| 12 months | -1.4 | 7.1 | -7.9 |  | -3.0 | 3.2 | -4.6 |
|  | -2.8, -0.1 | 0.5, 13.7 | -13.4, -2.3 |  | -4.2, -1.9 | -3.1, 9.5 | -11.4, 2.2 |
|  |  |  |  |  |  |  |  |
| Mean arterial pressure  (mmHg) |  |  |  |  |  |  |  |
| 6 months | 0.6 | -0.3 | 0.3 |  | -0.7 | -0.6 | -2.9 |
|  | -1.4, 2.5 | -2.4, 1.9 | -1.0, 1.7 |  | -1.5, 0.2 | -2.1, 0.8 | -3.6, -2.3 |
| 12 months | -1.7 | 4.3 | -9.0 |  | -1.8 | 2.2 | -11.8 |
|  | -3.9, 0.5 | 2.2, 6.4 | -12.0, -5.8 |  | -3.6, 0.0 | 0.9, 3.4 | -15.9, -7.7 |

Results are based on a mixed-model analysis using censored inverse probability weights.

^1^Delta: regression coefficient

^2^Adjusted for demographic, biological, behavioral, psychosocial, and work-related variables

***Hypertension (Table J)***

Between-group ITT analyses

Among workers in intervention companies with normal BP at baseline, BP changes were negative and large, ranging from -3.3 to -7.5 mmHg at 12 months, relative to those working in control companies. The subgroup with elevated BP at baseline experienced a BP reduction at 6 months of up to -6.7 mmHg in SBP (95% CI -10.4, -3.1) followed by a reversal of effect at 12 months (up to +8.4 mmHg in SBP (95% CI 1.3, 15.5). BP differences among workers with stage I hypertension (HT) at baseline were small and inconsistent. Among those with stage II HT, BP increases of up to +2.4 mmHg (95% CI -1.8, 6.7) in SBP were observed.

Within-group analyses

Participants with normal BP at baseline experienced substantial increases in BP, especially at 6 months, of up to +9.6 mmHg (95% CI 7.9, 11.0), with the exception of PP (see **Table J**).

The small group of 31 workers with elevated BP at baseline showed inconsistent BP changes (reductions of up to -16 mmHg in PP and increases of up to +10 mmHg in DBP), while participants with stage I and II HT experienced consistent BP reductions of approximately -2 mmHg and -10 mmHg, respectively, at both 6 and 12 months.

**Table J.** Between and within-group differences in blood pressure change from baseline to 6 and 12 months after intervention, stratified by baseline hypertension status, in the Mexican Institute of Social Security Study (N = 2,002).

|  | **Between-group (ITT)** | | | |  | **Within-group (Pre-post)** | | | |
| --- | --- | --- | --- | --- | --- | --- | --- | --- | --- |
|  | **Delta^1^ adjusted^2^** | | | |  | **Delta^1^ adjusted^2^** | | | |
|  | **Normal** | **Elevated** | **Stage I HT** | **Stage II HT** |  | **Normal** | **Elevated** | **Stage I HT** | **Stage II HT** |
|  | **(SBP<120 &**  **DBP<80)** | **(SBP:120-129 &**  **DBP<80)** | **(SBP:130-139 or**  **DBP:80-89)** | **(SBP:140-180 or**  **DBP:90-120)** |  | **(SBP<120 &**  **DBP<80)** | **(SBP:120-129 &**  **DBP<80)** | **(SBP:130-139 or**  **DBP:80-89)** | **(SBP:140-180 or**  **DBP:90-120)** |
|  | **n = 312** | **n = 69** | **n = 1,400** | **n = 221** |  | **n = 192** | **n = 31** | **n = 685** | **n = 103** |
| Systolic BP  (mmHg) |  |  |  |  |  |  |  |  |  |
| 6 months | 2.2 | -6.7 | 0.1 | 2.4 |  | 7.4 | -10.4 | -2.5 | -8.3 |
|  | -0.7, 5.0 | -10.4, -3.1 | -1.6, 1.8 | -1.8, 6.7 |  | 4.6, 10.2 | -14.5, -6.2 | -2.9, -2.0 | -12.7, -3.9 |
| 12 months | -7.5 | 8.4 | -0.5 | 1.8 |  | 0.3 | -1.7 | -3.0 | -10.8 |
|  | -11.5, -3.5 | 1.3, 15.5 | -1.7, 0.7 | -5.9, 9.6 |  | -2.9, 3.5 | -2.9, 0.4 | -3.6, -2.4 | -18.3, -3.3 |
| Diastolic BP (mmHg) | |  |  |  |  |  |  |  |  |
| 6 months | 2.6 | -2.4 | 0.1 | -0.5 |  | 9.6 | 6.1 | -1.8 | -9.5 |
|  | -0.5, 5.6 | -4.5, -0.2 | -1.7, 1.9 | -3.4, 2.3 |  | 7.6, 11.6 | 3.8, 8.4 | -2.0, -1.6 | -12.1, -6.9 |
| 12 months | -4.2 | 3.9 | 0.9 | -0.7 |  | 6.2 | 9.5 | -0.7 | -10.7 |
|  | -7.6, -0.8 | -0.9, 8.8 | 0.3, 1.6 | -5.0, 3.6 |  | 3.3, 9.1 | 7.9, 11.0 | -0.8, -0.6 | -14.8, -6.6 |
| Pulse pressure (mmHg) |  |  |  |  |  |  |  |  |  |
| 6 months | -0.4 | -4.3 | 0.1 | 2.5 |  | -2.2 | -16.4 | -0.6 | 0.9 |
|  | -2.5, 1.8 | -7.2, -1.4 | -0.5, 0.7 | 0.4, 4.6 |  | -4.3, 0.0 | -18.6, -14.2 | -1.3, 0.1 | -0.9, 2.8 |
| 12 months | -3.3 | 4.5 | -1.2 | 2.8 |  | -5.8 | -11.2 | -2.2 | 0.2 |
|  | -4.8, -1.8 | 1.3, 7.6 | -2.1, -0.2 | -2.7, 8.3 |  | -6.6, -5.1 | -12.4, -10.0 | -2.8, -1.6 | -3.0, 3.5 |
| Mean arterial pressure  (mmHg) |  |  |  |  |  |  |  |  |  |
| 6 months | 2.4 | -4.2 | 0.0 | 0.7 |  | 8.7 | -0.7 | -2.1 | -9.1 |
|  | -0.4, 5.2 | -6.7, -1.6 | -1.8, 1.8 | -2.6, 4.0 |  | 6.6, 10.8 | -3.8, 2.4 | -2.2, -1.9 | -12.3, -5.9 |
| 12 months | -5.6 | 5.7 | 0.4 | 0.5 |  | 3.7 | 4.8 | -1.6 | -10.7 |
|  | -9.2, -2.0 | 0.0, 11.4 | -0.4, 1.1 | -4.8, 5.7 |  | 0.7, 6.7 | 3.6, 6.0 | -1.9, -1.2 | -16.2, -5.2 |

Results are based on a mixed-model analysis using (non)censored inverse probability weights. ^1^

Delta: regression coefficient; ^2^Adjusted for demographic, biological, behavioral, psychosocial, and work-related variables

**7. Participation Rates by Baseline Levels of Cardiovascular Disease Risk Factors (Table K)**

**Table K.** Worker participation in offered intervention sessions by cardiovascular disease risk factor levels in intervention companies (n=1,011). Mexican Institute of Social Security 2009 (N = 2002).

|  | **Total intervention hours offered** | **Frequency** | **Maximum hours per group (if 100% compliance)** | **Total person-hours** | **Compliance (%)** |
| --- | --- | --- | --- | --- | --- |
| **Body mass index risk** |  |  |  |  |  |
| Underweight | 99 | 2 | 198 | 37 | 18.69 |
| Normal | 96 | 161 | 15456 | 2593 | 16.78 |
| Overweight | 99 | 219 | 21681 | 2564 | 11.83 |
| Obesity | 105 | 78 | 8190 | 948 | 11.58 |
| Extreme obesity | 117 | 4 | 468 | 75 | 16.03 |
| Total | 99 | 464 | 45993 | 6217 | 13.52 |
|  |  |  |  |  |  |
| **Income risk** |  |  |  |  |  |
| Low (<18K-54K) | 99 | 178 | 17622 | 2580 | 14.64 |
| Medium (54,001-162K) | 99 | 224 | 22176 | 2890 | 13.03 |
| High (162,001 - >198K) | 99 | 62 | 6138 | 747 | 12.17 |
| Total | 99 | 464 | 45936 | 6217 | 13.53 |
|  |  |  |  |  |  |
| **Glucose level risk** |  |  |  |  |  |
| Normal | 99 | 386 | 38214 | 5462 | 14.29 |
| Prediabetes | 99 | 65 | 6435 | 535 | 8.31 |
| Diabetes | 99 | 13 | 1287 | 220 | 17.09 |
| Total | 99 | 464 | 45936 | 6217 | 13.53 |
|  |  |  |  |  |  |
| **Blood pressure risk** |  |  |  |  |  |
| Normal  (SBP<120 & DBP<80) | 99 | 108 | 10692 | 1660 | 15.53 |
| Elevated  (SBP:120-129 & DBP<80) | 99 | 10 | 990 | 172 | 17.37 |
| Stage I HT  (SBP:130-139 or DBP:80-89) | 99 | 296 | 29304 | 3604 | 12.30 |
| Stage II HT  (SBP:140-180 or DBP:90-120) | 99 | 50 | 4950 | 781 | 15.78 |
| Total | 99 | 464 | 45936 | 6217 | 13.53 |

**8. Discussion of Supplementary Material**

Results shown in tables S1-S4 have been discussed in the main manuscript. The following discussion of results shown in supplemental tables S5-11 focuses on within-group analyses and analyses (between and within-group) stratified by intervention company, intervention program component, and individual particpant baseline level of selected cardiovascualr disease risk factors. While the main between group ITT analyses need to be considered primary for the overall evaluation of the effectiveness of our intervention, these supplemental analyses provide within-group pre-post data for comparison with the majority of WHP programs that have not used an ITT approach. In addition, the stratified analyses may be helpful for the design of future WHP intervention studies and the identifications of subpopulations that may benefit most from such interventions or from improved intervention strategies.

ITT analysis includes all workers assigned to an intervention, regardless of their adherence or subsequent withdrawal. This analysis avoids overestimation of favorable results obtained via removal of non-compliers and reflects more accurately the deviations from protocol and loss to follow-up occurring in real life ([1](#_ENREF_1)). Moreover, this approach prevents loss of statistical power due to exclusion of noncompliant subjects and dropouts; it also limits faulty inferences based on self-selected subgroups of participants (healthier subjects tend to participate more in preventive interventions ([1](#_ENREF_1), [2](#_ENREF_2))), and it emphasizes greater accountability for all subjects enrolled in the study. However, in ITT analysis, noncompliance tends to dilute the estimated treatment effect and results using this approach are generally conservative ([3](#_ENREF_3)).

Within-group analyses are often conducted because they remove subject variance from error terms used to test treatment effects; i.e., the subject serves as his/her own control and thus treatment differences are not confounded with subject differences (as long as within-group analyses are limited to participants evaluated pre and post intervention, see our respective sensitivity analyses **Tables S5 – S6**). However, within-subject analyses are also subject to contextual effects and violation of certain statistical assumptions that may bias and limit interpretation of results ([4](#_ENREF_4), [5](#_ENREF_5)).

**Within-group changes (overall and by intervention component, *cf.* Table E)**

As expected ([6](#_ENREF_6)), our within-group analyses showed consistent, negative BP changes at 6 months and even stronger effects at 12 months among intervention participants, with effect sizes up to five-fold larger than those found with between-group comparisons. In general, within-group changes restricted to participants with data at baseline and subsequent follow-up points in time reflect changes among the more enthusiastic and healthier participants ([2](#_ENREF_2)) and tend to substantially overestimate the average effects of an intervention in the entire target population as estimated by comparisons with control groups and using an ITT approach. However, other factors may influence both adherence and BP and could bias observed effects in both directions ([7](#_ENREF_7)).

Similar to our study, a randomized controlled trial (RCT) assessing the effect of different exercise programs in a community in London found reductions in SBP and DBP both evident in ITT between group and pre-post within-group analyses ([8](#_ENREF_8)). This RCT compared the effectiveness of a 10-week program of supervised exercise classes, two to three times a week in a local leisure center, and a 10-week instructor-led walking program, compared with a control group receiving only tailored advice and information on physical activity and local exercise facilities. The 943 patients who participated in this trial were aged between 40 and 74 years, were not physically active at the time of the intervention, and had at least one cardiovascular risk factor. BP differences between study groups at 10 weeks, 6 months, and one year were analyzed based on an ITT analysis adjusted for baseline values, age, and gender, and excluding people taking BP-lowering medication. Both ITT and within-group analyses revealed greatest BP reductions among the supervised leisure-center group at the one-year assessment; ITT: -5.4 mmHg in SBP (95% CI -7.74, -3.03) and -4.1 mmHg in DBP (95% CI -5.29, -2.82); within-group changes: -6.3 mmHg in SBP (95% CI -9.02, -3.64) and -4.3 mmHg in DBP (95% CI -5.73, -2.88).

***Within-group analyses stratified by company and intervention component (cf. Table F)***

Stratified analyses by company and intervention component revealed reductions in BP at 6 months, which were sustained and became even larger at 12 months. The strongest effects were observed for the stress management component of the intervention at the public health company, with reductions up to -6.8 mmHg (95% CI -10.9, -2.6) in PP and -6.5 mmHg (95% CI -11.6, -1.5) in SBP at 12 months. Data for the airline company at 12 months was not available because this company went bankrupt about one year after our study began, making it impossible to collect any data after our 6-month follow-up. Knowledge of imminent job loss and its associated anxiety may have been one of the reasons the stress component of the intervention did not seem to benefit workers, and even resulted in an increase of up to +1.6 mmHg in DBP at 6 months. Job insecurity has been associated with increased levels of BP due to the psychological strain over losing economic stability, facing family problems, and lacking control over one’s future ([9](#_ENREF_9)). In fact, a study in North Carolina reported a SBP average of 8 mmHg higher among men who reported a great deal of worry about losing their jobs compared to those who felt secure ([10](#_ENREF_10)). Individual worker behavioral stress management interventions may not be sufficient to reduce the strong deleterious health effects of such workplace organizational stressors and may need to be combined with primary prevention strategies as suggested in NIOSH’s Total Worker Health strategy ([11](#_ENREF_11)).

**Between and within-group analyses stratified by baseline cardiovascular risk factor levels (*cf.* Tables G – K)**

***Obesity***

In regards to obesity, people of normal BMI participated more often even though fewer sessions were offered to this subgroup of participants (*cf.* **Table K**). There is a decreasing rate of participation from low to high obesity risk. This pattern supports an old critique of workplace health promotion: highest participation rates and compliance are observed among those who need it the least ([12](#_ENREF_12)).

Moreover, the high-risk group of obese people in our study experienced no benefit from being in the intervention group. Among those who participated in program activities, we observed a reduction of up to -1.8 mmHg at six months, which was fully reversed at 12 months. It is unclear whether these results are due to the effects of a calorie-reduction hunger diet. A pattern known as ‘*yo-yo effect’* or ‘*weight cycling’* for temporary weight loss associated with calorie-restricted diets has been described in the obesity literature ([13](#_ENREF_13)) but to our knowledge, such an effect has not been reported for behavior-induced BP change. The mechanisms leading to this effect for the latter might be related to the job strain model of stress resulting from a combination of high demands combined with low control ([14](#_ENREF_14)): stress from hunger or impossible demands on behavior change combined with job, time and/or money constraints may lead to psychological strain that has been associated with high BP in the literature ([15](#_ENREF_15), [16](#_ENREF_16)). Further intervention studies with longer follow-up time are needed to investigate if such effect reversals are a typical limitation of behavioral change programs commonly used in workplace health promotion.

Additionally, obese BMI subgroups were offered and received considerable more intervention sessions than overweight or normal weight workers (as shown in **Table A**), which makes these strata results hard to compare directly. However, this difference in intervention intensity makes our findings even less supportive of the intervention because these obese workers who received the most nutrition sessions experienced no sustained benefit of BP reduction at all while normal and overweight workers attending fewer sessions experienced sustained BP reductions. Perhaps, attending nutrition sessions had un-intended negative effects for the high-risk group of obese workers. We are unaware of any reports of such negative effects, however, one possible explanation could be that obese participants who were unable to maintain weight loss may have experienced or feared reprimand from self or others in case they failed to follow the recommended dietary guidelines or to maintain any initial weight loss. In general, within-subject designs have been faulted for being subject to context effects of practice, sensitization, and carry-over ([4](#_ENREF_4)).

Another ten-week nutrition and physical activity worksite intervention also reported within-changes of approximately -2 mmHg in both SBP and DBP among participants at the end of the program, which did not differ among BMI risk categories. However, at one-year follow-up, SBP went back to baseline levels while DBP reductions were sustained, particularly in the obese category, confirming our observations ([17](#_ENREF_17)).

***Income***

Both the between and within-group analyses revealed BP reductions for the high-risk subgroups in this population (i.e., people with low or medium income levels). These reductions were sustained after one-year follow-up. This is an encouraging finding as research has shown that low-wage workers generally experience hardships that prevent them from benefiting from health promotion programs, such as financial and time constraints. Indeed, these workers are more likely to hold part-time, temporary, or multiple jobs. Also, this group is exposed to greater job insecurity and low organizational or managerial support, which may preclude them from participating in WHP programs ([18](#_ENREF_18)).

***Diabetes***

Our stratification by blood glucose levels showed consistent, large, and sustained reductions of BP (up to -14.0 mmHg in SBP at 12 months; 95% CI -16.7, -11.2) among diabetics, which indicates that this intervention might have been particularly beneficial for people in this high-risk subgroup.

About half of this working population with diabetes was newly diagnosed. Though these workers were given the recommendation at baseline to see their doctor, the data did not indicate whether this recommendation was followed. Therefore, we were unable to determine whether the lowering of BP resulted from the WHP intervention or from their primary physician who prescribed medication, or a combination of effects that we cannot separate in our data. Nevertheless, physician engagement could be one of the benefits of baseline blood glucose screening as part of the intervention, and could thus be one possible positive effect of this work intervention even though it had been neither hypothesized nor evaluated as such in our study. As diabetes and hypertension share etiology and disease mechanisms such as obesity, inflammation, oxidative stress, and insulin resistance ([19](#_ENREF_19)), people with hypertension have a higher risk of developing type 2 diabetes and vice-versa ([20](#_ENREF_20)), thus making screening a valuable tool to positively impact both conditions.

Although usual clinical care is heavily focused on these two CVD risk factors, it is evidently not sufficient for prevention. Health-promoting workplaces may help fill this gap, especially if they offer workplace health promotion using a Total Worker Health® approach as recommended by NIOSH. Such an approach would also include primary prevention of work-related physical and psychosocial job stressors that have been shown to increase BP, CVD, and mortality, such as prolonged standing ([21-23](#_ENREF_21)) heavy physical work ([24](#_ENREF_24), [25](#_ENREF_25)), noise ([26](#_ENREF_26)), lead ([27](#_ENREF_27)), toxic fumes and mineral dust ([28](#_ENREF_28), [29](#_ENREF_29)), environmental heat stress ([30](#_ENREF_30)), effort-reward imbalance ([31](#_ENREF_31)), and job strain ([32](#_ENREF_32)); factors that have not been addressed in the evaluated intervention.

The prevalence of diabetes mellitus (DM) is rapidly increasing on a global scale. Mexico is one of the countries in which such precipitous growth has been observed. The proportion of inhabitants with diagnosed DM increased four-fold from 1993 to 2006, directly affecting about a quarter of the population. The impact of DM on overall mortality increased by over 20 times in the same 13-year period, and future projections see this estimate increasing further. In 2011, health spending attributed to diabetes in the country amounted to almost eight billion dollars. A large portion of this spending is in the form of out-of-pocket expenses, largely affecting the uninsured population the most ([33](#_ENREF_33)).

As government expenditure on health continues to decrease, it is paramount that Mexican institutions allocate monetary resources efficiently in the areas where most benefit will be achieved ([34](#_ENREF_34)). This study indicates that developing worksite health promotion programs directed to reach high-risk DM populations may also be favorable to reduce BP and prevent complications arising from these two diseases.

***Hypertension***

ITT analyses revealed that participants with normal BP seemed to benefit more from the intervention, while the high-risk groups with different stages of manifest hypertension showed relative increases in BP among workers in intervention companies compared to those in control companies. In contrast, within-group analyses showed consistent BP reductions (up to -10.8 mmHg (95% CI -18.3, -3.3) in SBP at 12 months among people with Stage II HT) except for the subgroup with normal BP at baseline. On the one hand, our findings resulting from our ITT analyses are not consistent with extant studies. For example, a RCT found -7.5 mmHg SBP (95% CI -14.7, -0.2) and -3.1 mmHg DBP (95% CI -7.1, 1.0) difference between intervention and control companies using an ITT approach among 92 participants with hypertension at baseline after a stress and anger management intervention. However, the follow-up period of this study was only 10 weeks and sustainability of results over a longer period is unknown ([35](#_ENREF_35)). On the other hand, similar to our findings, studies using a within-subject design have reported reductions in BP among individuals with high risk of HT or with HT at baseline. For example, a six-year health promotion program in a Malaysian public university resulted in a reduction of -0.1 and -0.2 mmHg per year in SBP and DBP, respectively. After stratifying by subgroups of hypertension, researchers of this study reported a reduction of -2 mmHg per year among subgroups with hypertension and at-risk of hypertension (the authors defined the subgroups as follows: “Hypertension” – self-reported clinical diagnosis of HT, with or without medication use; “At-risk” – no self-reported medical history of HT, yet SBP ≥ 120mmHg and/or DBP ≥ 80 mmHg at baseline; and “Healthy” – no self-reported medical history of HT and SBP < 120 mmHg and DBP < 80 mmHg at baseline) ([36](#_ENREF_36)). Another one-year intervention program evaluating within-subject changes in several health outcomes and including nutrition, exercise, sleep, and stress management components reported a -7% reduction post-intervention of borderline/high BP, which was determined by repeated answers to the following question: “Have you ever been told by a doctor that you have high BP? Yes; Yes, but female told only during pregnancy; No; No, pre-HT or borderline HT; Don’t know/Not sure; Refused” ([37](#_ENREF_37)). Finally, reductions of up to -12 mmHg in both SBP and DBP were observed after participation in a physical activity intervention program among workers with baseline risk for hypertension. However, in this study the intervention only lasted 12 weeks ([38](#_ENREF_38)).

**9. References**

1. McCoy CE. Understanding the intention-to-treat principle in randomized controlled trials. The Western Journal of Emergency Medicine. 2017;18(6):1075-8. doi:

2. Shrank WH, Patrick AR, Brookhart MA. Healthy user and related biases in observational studies of preventive interventions: a primer for physicians. J Gen Intern Med. 2011;26(5):546-50. doi: 10.1007/s11606-010-1609-1

3. Gupta SK. Intention-to-treat concept: A review. Perspectives in clinical research. 2011;2(3):109-12. doi: 10.4103/2229-3485.83221

4. Greenwald AG. Within-subjects designs: To use or not to use? Psychol Bull. 1976;83(2):314-20. doi:

5. Charness G, Gneezy U, Kuhn MA. Experimental methods: Between-subject and within-subject design. Journal of Economic Behavior & Organization. 2012;81(1):1-8. doi:

6. Salkind NJ. Within-subjects design. In: Salkind NJ, editor. Encyclopedia of research design. 3. Thousand Oaks, California: SAGE Publishing; 2010. doi: <https://dx.doi.org/10.4135/9781412961288.n503>

7. Weiss NS. Clinical Epidemiology. In: Rothman KJ, Greenland S, Lash TL, editors. Modern Epidemiology. Third ed. Philadelphia USA: Lippincott Williams & Wilkins; 2012. p. 641-51. doi:

8. Isaacs AJ, Critchley JA, Tai SS, Buckingham K, Westley D, Harridge SD, et al. Exercise Evaluation Randomised Trial (EXERT): a randomised trial comparing GP referral for leisure centre-based exercise, community-based walking and advice only. Health technology assessment (Winchester, England). 2007;11(10):1-165, iii-iv. doi: 10.3310/hta11100

9. Kalil A, Ziol-Guest KM, Hawkley LC, Cacioppo JT. Job insecurity and change over time in health among older men and women. J Gerontol B Psychol Sci Soc Sci. 2010;65b(1):81-90. doi: 10.1093/geronb/gbp100

10. James SA, LaCroix AZ, Kleinbaum DG, Strogatz DS. John Henryism and blood pressure differences among black men. II. The role of occupational stressors. J Behav Med. 1984;7(3):259-75. doi: 10.1007/bf00845359

11. National Institute for Occupational Safety and Health. What is Total Worker Health? Washington, D.C.: U.S. Department of Health & Human Services; 2018 [Available from: <https://www.cdc.gov/niosh/twh/totalhealth.html>. doi:

12. Lewis RJ, Huebner WW, Yarborough CM, 3rd. Characteristics of participants and nonparticipants in worksite health promotion. Am J Health Promot. 1996;11(2):99-106. doi: 10.4278/0890-1171-11.2.99

13. Rhee EJ. Weight Cycling and Its Cardiometabolic Impact. Journal of obesity & metabolic syndrome. 2017;26(4):237-42. doi: 10.7570/jomes.2017.26.4.237

14. Karasek RA, Jr. Job demands, job decision latitude, and mental strain: implications for job redesign. Admin Sci Quart. 1979;24(2):285-308. doi:

15. Schnall PL, Schwartz JE, Landsbergis PA, Warren K, Pickering TG. A longitudinal study of job strain and ambulatory blood pressure: results from a three-year follow-up. Psychosom Med. 1998;60(6):697-706. doi:

16. Feaster M, Krause N. Job strain associated with increases in ambulatory blood and pulse pressure during and after work hours among female hotel room cleaners. Am J Ind Med. 2018;61(6):492-503. doi: 10.1002/ajim.22837

17. Thorndike AN, Healey E, Sonnenberg L, Regan S. Participation and cardiovascular risk reduction in a voluntary worksite nutrition and physical activity program. Prev Med. 2011;52(2):164-6. doi: 10.1016/j.ypmed.2010.11.023

18. Stiehl E, Shivaprakash N, Thatcher E, Ornelas IJ, Kneipp S, Baron SL, et al. Worksite Health Promotion for Low-Wage Workers: A Scoping Literature Review. Am J Health Promot. 2018;32(2):359-73. doi: 10.1177/0890117117728607

19. Cheung BM, Li C. Diabetes and hypertension: is there a common metabolic pathway? Current atherosclerosis reports. 2012;14(2):160-6. doi: 10.1007/s11883-012-0227-2

20. Emdin CA, Anderson SG, Woodward M, Rahimi K. Usual Blood Pressure and Risk of New-Onset Diabetes: Evidence From 4.1 Million Adults and a Meta-Analysis of Prospective Studies. Journal of the American College of Cardiology. 2015;66(14):1552-62. doi: 10.1016/j.jacc.2015.07.059

21. Smith P, Ma H, Glazier RH, Gilbert-Ouimet M, Mustard C. The Relationship Between Occupational Standing and Sitting and Incident Heart Disease Over a 12-Year Period in Ontario, Canada. Am J Epidemiol. 2018;187(1):27-33. doi: 10.1093/aje/kwx298

22. Krause N, Lynch JW, Kaplan GA, Cohen RD, Salonen R, Salonen JT. Standing at work and progression of carotid atherosclerosis. Scand J Work Environ Health. 2000;26(3):227-36. doi: 10.5271/sjweh.536

23. Hall C, Heck JE, Sandler DP, Ritz B, Chen H, Krause N. Occupational and leisure-time physical activity differentially predict 6-year incidence of stroke and transient ischemic attack in women. Scand J Work Environ Health. 2018. doi: 10.5271/sjweh.3787

24. Coenen P, Huysmans MA, Holtermann A, Krause N, van Mechelen W, Straker LM, et al. Do highly physically active workers die early? A systematic review with meta-analysis of data from 193 696 participants. British Journal of Sports Medicine. 2018;52(20):1320. doi:

25. Holtermann A, Krause N, van der Beek AJ, Straker L. The physical activity paradox: six reasons why occupational physical activity (OPA) does not confer the cardiovascular health benefits that leisure time physical activity does. Br J Sports Med. 2018;52(3):149-50. doi: 10.1136/bjsports-2017-097965

26. Hahad O, Kroller-Schon S, Daiber A, Munzel T. The Cardiovascular Effects of Noise. Deutsches Arzteblatt international. 2019;116(14):245-50. doi: 10.3238/arztebl.2019.0245

27. Kosnett MJ, Wedeen RP, Rothenberg SJ, Hipkins KL, Materna BL, Schwartz BS, et al. Recommendations for medical management of adult lead exposure. Environ Health Perspect. 2007;115(3):463-71. doi: 10.1289/ehp.9784

28. Uzoigwe JC, Prum T, Bresnahan E, Garelnabi M. The emerging role of outdoor and indoor air pollution in cardiovascular disease. North American journal of medical sciences. 2013;5(8):445-53. doi: 10.4103/1947-2714.117290

29. Zaky A, Ahmad A, Dell'Italia LJ, Jahromi L, Reisenberg LA, Matalon S, et al. Inhaled matters of the heart. Cardiovascular regenerative medicine. 2015;2. doi: 10.14800/crm.997

30. Crandall CG, Gonzalez-Alonso J. Cardiovascular function in the heat-stressed human. Acta physiologica (Oxford, England). 2010;199(4):407-23. doi: 10.1111/j.1748-1716.2010.02119.x

31. Siegrist J. Effort-reward imbalance at work and cardiovascular diseases. Int J Occup Med Environ Health. 2010;23(3):279-85. doi: 10.2478/v10001-010-0013-8

32. Kivimaki M, Nyberg ST, Batty GD, Fransson EI, Heikkila K, Alfredsson L, et al. Job strain as a risk factor for coronary heart disease: a collaborative meta-analysis of individual participant data. Lancet. 2012;380(9852):1491-7. doi: 10.1016/S0140-6736(12)60994-5

33. Arredondo A, Reyes G. Health disparities from economic burden of diabetes in middle-income countries: evidence from Mexico. PLoS One. 2013;8(7):e68443. doi: 10.1371/journal.pone.0068443

34. Arredondo A, Orozco E, Alcalde-Rabanal J, Navarro J, Azar A. Challenges on the epidemiological and economic burden of diabetes and hypertension in Mexico. Revista de saude publica. 2018;52:23. doi: 10.11606/s1518-8787.2018052000293

35. Clemow LP, Pickering TG, Davidson KW, Schwartz JE, Williams VP, Shaffer JA, et al. Stress management in the workplace for employees with hypertension: a randomized controlled trial. Translational behavioral medicine. 2018;8(5):761-70. doi: 10.1093/tbm/iby018

36. Eng JY, Moy FM, Bulgiba A. Impact of a Workplace Health Promotion Program on Employees' Blood Pressure in a Public University. PLoS ONE. 2016;11(2):e0148307. doi:

37. Merrill RM, Anderson A, Thygerson SM. Effectiveness of a worksite wellness program on health behaviors and personal health. J Occup Environ Med. 2011;53(9):1008-12. doi: 10.1097/JOM.0b013e3182281145

38. Corbett DB, Fennell C, Peroutky K, Kingsley JD, Glickman EL. The effects of a 12-week worksite physical activity intervention on anthropometric indices, blood pressure indices, and plasma biomarkers of cardiovascular disease risk among university employees. BMC research notes. 2018;11(1):80. doi: 10.1186/s13104-018-3151-x
